# Subcortical functional connectivity gradients in temporal lobe epilepsy

**DOI:** 10.1016/j.nicl.2023.103418

**Published:** 2023-05-05

**Authors:** Alfredo Lucas, Sofia Mouchtaris, Eli J. Cornblath, Nishant Sinha, Lorenzo Caciagli, Peter Hadar, James J. Gugger, Sandhitsu Das, Joel M. Stein, Kathryn A. Davis

**Affiliations:** a Perelman School of Medicine, University of Pennsylvania, United States; b Department of Bioengineering, University of Pennsylvania, United States; c Department of Neurology, University of Pennsylvania, United States; d Department of Neurology, Massachusetts General Hospital, United States; e Department of Radiology, University of Pennsylvania, United States

**Keywords:** Subcortex, Focal epilepsy, Dimensionality, Connectome

## Abstract

**Background and Motivation:**

Functional gradients have been used to study differences in connectivity between healthy and diseased brain states, however this work has largely focused on the cortex. Because the subcortex plays a key role in seizure initiation in temporal lobe epilepsy (TLE), subcortical functional-connectivity gradients may help further elucidate differences between healthy brains and TLE, as well as differences between left (L)-TLE and right (R)-TLE.

**Methods:**

In this work, we calculated subcortical functional-connectivity gradients (SFGs) from resting-state functional MRI (rs-fMRI) by measuring the similarity in connectivity profiles of subcortical voxels to cortical gray matter voxels. We performed this analysis in 24 R-TLE patients and 31 L-TLE patients (who were otherwise matched for age, gender, disease specific characteristics, and other clinical variables), and 16 controls. To measure differences in SFGs between L-TLE and R-TLE, we quantified deviations in the average functional gradient distributions, as well as their variance, across subcortical structures.

**Results:**

We found an expansion, measured by increased variance, in the principal SFG of TLE relative to controls. When comparing the gradient across subcortical structures between L-TLE and R-TLE, we found that abnormalities in the ipsilateral hippocampal gradient distributions were significantly different between L-TLE and R-TLE.

**Conclusion:**

Our results suggest that expansion of the SFG is characteristic of TLE. Subcortical functional gradient differences exist between left and right TLE and are driven by connectivity changes in the hippocampus ipsilateral to the seizure onset zone.

## Highlights

• First principal subcortical gradient follows lower to higher computational hierarchy.
• Patients with temporal lobe epilepsy (TLE) have an expanded first principal gradient.
• The ipsilateral hippocampus drives expanded principal gradient in right vs. left TLE.

## 1 Introduction

Epilepsy is a brain disorder characterized by recurrent seizures that result from abnormal synchronization of neural activity. While seizures are well controlled with anti-seizure medications in many individuals with epilepsy, nearly a third of them are pharmacoresistant (Kwan and Brodie, 2000). Many of these cases occur in patients with temporal lobe epilepsy (TLE), the most common type of focal epilepsy (Querol Pascual, 2007). In these patients, seizures originate in the mesial temporal lobe (hippocampus, parahippocampal gyrus, and amygdala) or in the temporal neocortex. In unilateral TLE, seizures can be localized to either the left (L-TLE) or the right temporal lobe (R-TLE). Initially, L-TLE and R-TLE were thought to be symmetric disorders, but recent work has provided evidence to the contrary (Lemkaddem et al., 2014, Voets et al., 2012, Zhang et al., 2010, Voets et al., 2011). However, current literature disagrees as to whether L- or R-TLE has more extensive abnormalities (Caciagli et al., 2014).

Epilepsy is being increasingly conceptualized as a network disorder, with abnormal connections across the brain (Kramer and Cash, 2012, Bassett and Bullmore, 2006). These abnormalities can be quantified and compared across patients via the application of graph-theoretical approaches to measures of functional connectivity, generated from functional MRI (fMRI) (Lee et al., 2013, Xiao et al., 2017, Bullmore and Sporns, 2009). In TLE, these approaches have demonstrated both local and global epileptic network abnormalities despite focal seizure localization (Fadaie et al., 2021, Pedersen et al., 2015, Trimmel et al., 2021).

Functional connectivity matrices can additionally be used to generate functional connectivity gradients, using linear and non-linear dimensionality reduction techniques (Haak et al., 2018). Functional connectivity gradients provide a simple, yet efficient, representation of connectivity across the brain, where regions close to each other in gradient space have similar connectivity profiles (Bernhardt et al., 2022). Initial work demonstrated that the first principal cortical gradient anchors unimodal sensory and motor regions at one end, and transmodal regions involved in higher level processing, such as those belonging to frontoparietal and default mode networks at the opposite end. In addition, the second principal gradient can differentiate between visual and somatosensory/motor regions (Margulies et al., 2016). These functional gradients are altered in disease states (Park et al., 2021, Meng et al., 2021, Dong et al., 2020). In idiopathic generalized epilepsy, the values taken by the first gradient are expanded, suggestive of increased differentiation in connectivity profiles compared to controls (Meng et al., 2021). In TLE, a similar functional connectivity distance metric (distinct from gradients) was also found to be contracted in temporoinsular and prefrontal networks relative to controls (Larivière et al., 2020). Functional activation derived from task-based fMRI compared to resting state functional gradients probe functional reorganization due to cognitive impairment in epilepsy (Fadaie et al., 2021, Caciagli, xxxx). In subcortical-to-cortical functional connectivity gradients, subcortical voxels/regions with similar connections to cortical gray matter regions will be close together in gradient space. These gradients were used to elucidate the topographical organization of the subcortex (Tian et al., 2020), and demonstrate a unimodal to transmodal organization in the thalamus similar to that in the cortex (Meng et al., 2021, Yang et al., 2020). To our knowledge, no work has explored the effects of epilepsy on subcortical-to-cortical functional gradients, despite the prominent role of subcortical structures in the pathophysiology of epilepsy.

In this study, we use subcortical functional connectivity gradients generated from 3 T resting-state fMRI to compare TLE to healthy controls, and to investigate patterns of reorganization that may be specific to L-TLE and R-TLE. We tested whether the first principal gradient was expanded in TLE as compared to healthy controls, in line with prior work (Fadaie et al., 2021, Meng et al., 2021). We also tested whether gradient distributions in key subcortical structures that contribute to seizures were altered in patients with TLE. Lastly, we explored whether changes seen in L-TLE differed from those seen in R-TLE, which would further contribute to the evidence in favor of key functional connectome differences between the two disease states.

## 2 Methods

### 2.1 Patient Demographics

Data acquisition for this study was approved by the institutional review board of the University of Pennsylvania. A total of 55 temporal lobe epilepsy (TLE) patients were included in this study. Localization of seizure focus was determined during the Penn Epilepsy Surgical Conference (PESC) following evaluation of various clinical, neuroimaging, and neurophysiological data including: seizure semiology, neuropsychological testing, MRI, positron emission tomography (PET), scalp EEG, and intracranial EEG findings. Single subject clinical characteristics, including: whether intracranial EEG was used in the decision-making process, the lateralization of the PET hypometabolism, and the seizure surgical outcome (where available) are included in Supplementary Table 1. Thirty-one patients had left-sided seizure onset zone (SOZ) lateralization (L-TLE), and 24 patients had right-sided SOZ lateralization (R-TLE). Age, gender, disease duration, presence of MTS, and history of focal to bilateral tonic-clonic seizures (BTCS) are reported in Table 1**.** Lateralization for non-lesional subjects was determined by a combination of intracranial electrophysiology (iEEG; where available), and PET hypometabolism in combination with clinical semiology and EMU scalp EEG findings (if iEEG was not available). We found no statistically significant differences in demographic variables between the L-TLE and R-TLE groups. Sixteen age and gender matched controls (mean age 32 ± 11) were also included in the study.

**Table 1:** **Subject Demographics:** Table demographics showing number of subjects, age, sex, disease duration in years, presence of mesial temporal sclerosis (MTS), and history of focal to bilateral tonic-clonic seizures (BTCS).

**Subject Demographics**			
**Characteristic**	Group		
	**L-TLE**	**R-TLE**	**Control**
**Number of Subjects**	31	24	16
**Age**	35 ± 10	39 ± 12	32 ± 11
**Female**	16	12	7
**Disease Duration (years)**	16 ± 13	17 ± 15	**–**
**Clinical Characteristics**			
*MTS*	6	9	**–**
*BTCS History*	23	16	**–**

### 2.2 Image acquisition

For 46/55 TLE patients and 8/16 controls, we used a Siemens 3 T Magnetom PrismaFit scanner. rs-fMRI data were acquired during a 9-min interval with an axial, 72-slice gradient echo-planar sequence, TE/TR = 37/800 ms, with a 2 mm isotropic voxel size (protocol 1). For the remaining 9/55 TLE patients and 8/16 controls of the Penn cohort, we used a Siemens 3 T Magnetom Trio scanner. For this subset of patients, resting-state fMRI data were acquired during a 6-min interval with an axial, 72-slice gradient echo-planar sequence, TE/TR = 37/800 ms, with a 2 mm isotropic voxel size (protocol 2). High-resolution T1-weighted images, with a sagittal, 208-slice MPRAGE sequence, TE/TR = 2.24/2400 ms, with a 0.8 mm isotropic voxel size were acquired in all participants.

### 2.3 Neuroimaging processing

We used fMRIPrep (Esteban et al., 2019) to perform brain extraction and segmentation of individual T1-weighted (T1w) images, registration of rs-fMRI data to individual T1w and MNI template space, and time-series confound estimation. We used the fMRIPrep output data as our input to the xcpEngine post-processing pipeline for 36-parameter confound regression, demeaning, detrending and temporal filtering (Ćirić et al., 2020). Complete details about the functional and anatomical processing pipelines can be found in previous work (Lucas et al., 2023).

### 2.4 Subcortical gradient calculation

**Fig. 1:**
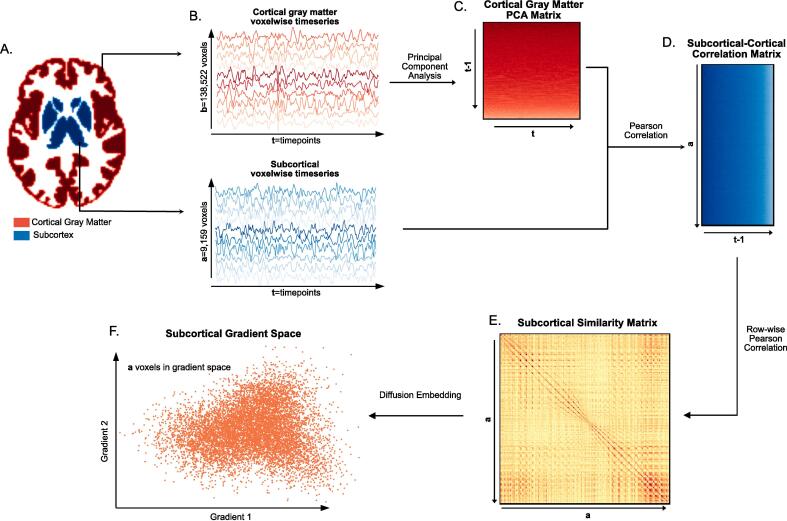
**Subcortical Functional Gradient Generation**: For each subject, **A.** the subcortical voxels and the cortical gray matter voxels were identified using the Harvard-Oxford subcortical and cortical atlases respectively, and **B.** the timeseries in each of these voxels was extracted. To reduce the computational complexity of the gradient estimation, **C.** the PCA of the cortical gray matter timeseries was taken, resulting in a cortical gray matter PCA matrix. **D.** The Pearson correlation between the cortical gray matter PCA matrix and the subcortical timeseries matrix was computed, resulting in a subcortical-cortical correlation matrix. **E.** The subcortical similarity matrix was estimated by computing the row-wise Pearson correlation of the subcortical-cortical correlation matrix, and **F.** diffusion embedding was applied to the subcortical similarity matrix resulting in the gradient space representation of the subcortical voxels.

Using the Harvard-Oxford cortical and subcortical atlases, we separated the fMRI time-series data into subcortical voxels [*t* seconds × *a* voxels] and cortical grey matter voxels [*t* seconds × *b* voxels] (Fig. 1A,B). In our dataset *a = 9,159*, *b = 138,522* and *t = 675* for protocol 1 and *t = 450* for protocol 2. For computational reasons, and given that there were much fewer timepoints than there were cortical gray matter voxels (*t*≪*b*), the cortical grey matter time-series data was reduced using principal component analysis (PCA) to a dimensionality of [*t* × *t-1]*, following an approach analogous to Haak et al., (Haak et al., 2018) (Fig. 1C). We then computed the Pearson correlation of every subcortical voxel’s time-series to each principal component, resulting in a [*a* voxels × *t-1*] matrix containing the functional connectivity of each subcortical voxel to each cortical voxel in principal component space (Fig. 1D). This resulting functional connectivity matrix was thresholded with only the top 10% of connections in each row remaining (Meng et al., 2021). The remaining steps of the gradient calculations were then completed using the BrainSpace Toolbox (Vos de Wael et al., 2020). First, the functional connectivity matrix was transformed into a similarity matrix by taking the Pearson correlation between every pair of rows [*a* voxels × *a* voxels] (Fig. 1D). Then, the gradients were generated using diffusion map embedding (Fig. 1E). For computational considerations, only the first 250 gradients were generated.

Due to differences in gradient ordering and signs, the gradients needed to be aligned to a template before comparison. Following Hong et al., (Hong et al., 2020), we horizontally stacked the gradients of all 71 participants (patients + controls) [*a* voxels by 250 * 71]. Using PCA, the first 250 principal components were generated [*a* voxels × 250]. The gradients of all patients were aligned to this template using a Procrustes alignment (Schönemann, 1966), which utilizes linear transformations (i.e. translation, scaling, rotation) to align a source to a target.

To account for differences in fMRI collection protocols, the resulting aligned gradients were harmonized using NeuroCombat (Fortin et al., 2018). This process regressed out differences in gradients due to protocol, but preserved differences due to seizure lateralization and control status.

### 2.5 Gradient estimation stability

To assess the stability of the results, we computed the correlation between the gradients obtained in our original approach, with the gradients obtained through different correlation techniques for similarity matrix calculation (the step between Fig. 1D and Fig. 1E), and different embedding techniques for gradient calculation (the step between Fig. 1E and Fig. 1F). In our approach proposed above, the similarity matrix was calculated using Pearson’s correlation coefficient, so we also investigated the use of 5 alternative measures: Gaussian kernels, cosine similarity, normalized angle similarity, and Spearman rank order correlations. Additionally, the gradients were originally calculated using diffusion maps, so we considered the use of Laplacian eigenmaps and PCA. This yielded (5x3 = ) 15 different gradient variants.

To compare the gradients estimated by different methods, we averaged together the aligned and harmonized first and second gradients of all patients for each method, and computed the magnitude of the Pearson correlation between every pair of gradients. The magnitude was used because the gradients within a single method were aligned to each other but not to the gradients of other methods, so the possibility of the gradients having inverted signs had to be accounted for.

### 2.6 Statistical analysis

The eigenvalue associated with each gradient gives a measure of relative importance (Porte et al., 2008). Thus, in line with prior work (Margulies et al., 2016, Meng et al., 2021), only the first two gradients with the largest eigenvalues were considered in subsequent analyses (eigenvalue 1 = 0.18 ± 0.04, and eigenvalue 2 = 0.14 ± 0.02). The eigenvalues of gradients 1–5 are shown in Supplementary Fig. 1. No differences were found between the eigenvalues of controls, L-TLE and R-TLE across gradients.

The statistics of interest were the mean and variance of the gradient value within each ROI, as well as the variance across the whole gradient. We use variance as an estimation of gradient expansion and contraction, in line with prior work (Meng et al., 2021, Dong et al., 2020). The comparison of individual gradient variance is equivalent to computing the univariate dispersion, described in prior work (Park et al., 2021, Bethlehem et al., 2020), for each gradient. We opted for using the variance due to its ease of interpretation and because it operationalizes some of the qualitative analyses done in prior work when comparing distributions of gradients (Meng et al., 2021, Dong et al., 2020). Finally, we note that because the Procrustes Alignment ensures that the gradients are on the same scale, the variance or dispersion of individual or group level gradients can be readily compared without concern for scaling effects.

Given the first and second principal gradients for each patient, we compared differences between groups in two ways: in average gradient space, and in subject space. For the average gradient space comparison, a voxel-wise average was taken across all subjects in each group, yielding a single representative gradient distribution for each group. In the subsequent analyses, we compared regions ipsilateral and contralateral to the SOZ in one group, with the corresponding ipsilateral and contralateral regions in the other group. Since left and right ROIs were flipped as necessary, both L-TLE and R-TLE had an ipsilateral and a contralateral ROI for comparison. With left-to-right flipping, the left sided ROIs in the L-TLE group were always compared with the right sided ROIs in the R-TLE group. To ensure that the measured differences were not driven by inherent asymmetries between the left and right ROIs, we randomly shuffled the group assignments and computed the statistics of interest between the ROIs in the shuffled groups. This procedure was repeated 2000 times, generating a null distribution for each statistic of interest. The p-value (p_PERM_) was determined by the fraction of the null distribution that exceeds the true value in a two-tailed fashion. Additionally, where indicated, a Bonferroni procedure was used to correct for multiple comparisons (p_BON_).

For the subject space comparison, the same statistics of interest (mean and variance) were computed for each individual patient, and the distribution of the statistics of interest was compared across groups. In this case, to ensure that the differences were not driven by inherent asymmetries, the subject space distributions were z-scored relative to the distribution of controls across left and right ROIs rather than ipsilateral and contralateral ROIs (i.e. in L-TLE, the left ROIs were z-scored to the left ROIs in controls, and in R-TLE, the right ROIs regions were z-scored to the right ROIs in controls). T-tests were computed based on these z-scored distributions.

In a subsequent average gradient space analysis, we considered the first and second principal gradients simultaneously to determine whether similar differences could be captured. The distribution spanned by principal gradients 1 and 2 was approximately normal and was estimated as a multivariate normal distribution with a [2 × 1] μ and [2 × 2] Σ. We compared the gradients in ipsilateral and contralateral regions between R-TLE and L-TLE using the Bhattacharyya distance, a metric for measuring the similarity of two multivariate normal distributions. To estimate the significance of the Battacharyya distance, we used the same permutation procedure described above.

### 2.7 Linear model for clinical variables

In addition to studying the effect of disease laterality on the gradient mean and variance, we also measured the effect of clinical variables on these two metrics. To do so, we used a linear model defined as:

$$y=\beta_{0}+\beta_{\mathrm{lat}}X_{\mathrm{lat}}+\beta_{\mathrm{MTS}}X_{\mathrm{MTS}}+\beta_{\mathrm{BTCS}}X_{\mathrm{BTCS}}+\beta_{\mathrm{duration}}X_{\mathrm{duration}}$$

where y is either the subject level mean or variance of the gradient, $X_{\mathrm{lat}}$ is an indicator for laterality (L-TLE vs. R-TLE), $X_{\mathrm{MTS}}$ is an indicator variable for the presence of mesial temporal sclerosis (MTS) as determined by structural imaging, $X_{\mathrm{BTCS}}$ is an indicator variable for the presence of a history of bilateral tonic-clonic seizures, and $X_{\mathrm{duration}}$ is a continuous variable that represents the number of years a subject has had epilepsy for. The above model was applied for each subcortical ROI.

## 3 Results

### 3.1 Subcortical functional connectivity gradients follow a lower to higher computational hierarchy along the first principal gradient

**Fig. 2:**
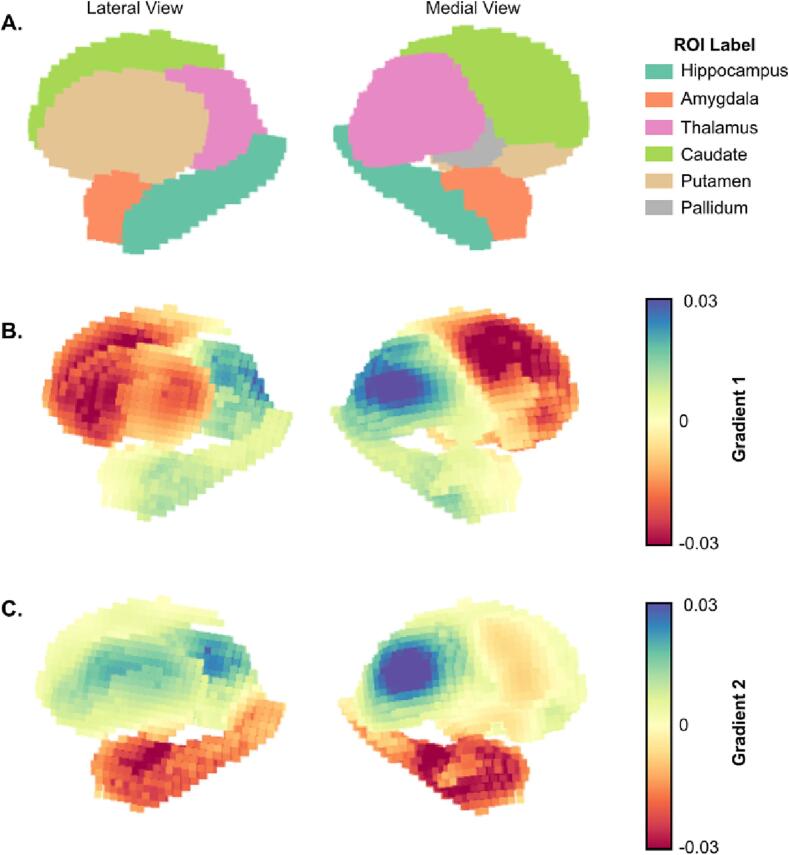
**Gradient projection on the subcortical surface: A.** Lateral (left) and medial (right) view of subcortical regions of interest (ROI) with corresponding labels. Subcortical surface projection of the average across all TLE subjects for **B.** gradient 1 and **C.** gradient 2.

**Fig. 3:**
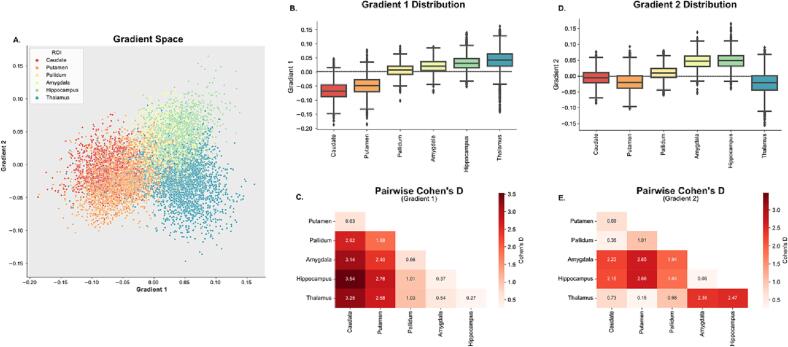
**Overview of the Subcortical Functional Gradients: A.** Average gradient space generated by principal gradient 1 and 2 across all TLE subjects. Different colors represent different subcortical regions of interest (ROIs). Ipsilateral and contralateral structures are assigned the same color in this representation. **B.,C.** Boxplots representing the distribution across ROIs for gradient 1 (**B.**) and gradient 2 (**C.**). **D.**, **E.** Pairwise Cohen’s D values between each ROI for gradient 1 (**D.**) and gradient 2 (**E.**). Differences between all ROIs were statistically significant (p_BON_ < 0.05).

We estimated the functional connectivity signature across subcortical voxels of TLE patients by computing their functional connectivity gradients from subcortical voxels to cortical gray matter voxels. The first two principal gradient values for each subcortical ROI resulted in separate functional clusters, mostly following the anatomical organization of the subcortex (Fig. 2, Fig. 3A), and this gradient organization was highly consistent across different gradient estimation approaches (Supplementary Figure 10). Interestingly, voxels within functionally related, but anatomically separate regions, such as the caudate and the putamen, had substantial overlap in gradient space. Further dividing each ROI into ipsilateral and contralateral demonstrates that ipsilateral ROIs have similar gradient distributions as their contralateral counterparts (Supplementary Fig. 3). In controls, left and right ROIs also had very similar gradient distributions, with no significant differences between the left–right hippocampus, left–right caudate and left–right pallidum, and significant but small (Cohen’s *d* < 0.5) differences between left–right thalamus, amygdala and putamen (Supplementary Fig. 4).

Mean values across gradient 1 suggest an organization that progresses from regions with lower computational hierarchy (with neural signaling that primarily projects to the thalamus) to regions with higher computational hierarchy (hippocampus and thalamus) (Fig. 3B). This is similar to Margulies et. al.’s work in cortical functional gradients, which demonstrated a unimodal to transmodal organization along the first principal gradient (Hong et al., 2019). These results were also replicated in the control cohort (Supplementary Fig. 5). All mean ROI pairwise differences across gradient 1 were significant (p < 0.001, Bonferroni corrected). However, pairwise Cohen’s D effect sizes between ROIs (Fig. 3C) were smallest between the caudate and putamen, as well as between the thalamus, hippocampus and amygdala. The proximity along the dimension of gradient 1 for the hippocampus and amygdala relative to the thalamus was countered by separation along the dimension of gradient 2 (Fig. 3D,E), suggesting that gradient 2 represents a separation between functions within lower and higher computational hierarchy domains (e.g. the thalamus is functionally different from the hippocampus and the amygdala, but they are both of high computational hierarchy).

### 3.2 Subcortical principal gradient 1 is expanded in temporal lobe epilepsy

**Fig. 4:**
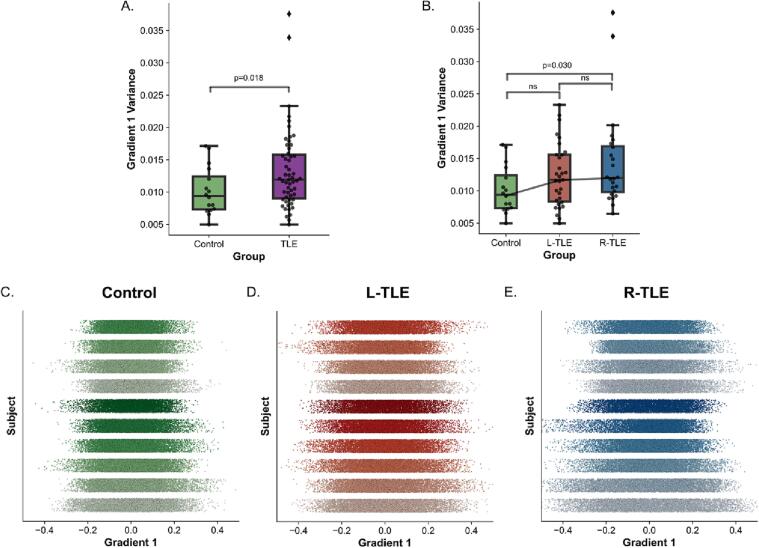
**Increased Gradient 1 Global Variance in TLE:** Boxplot for global gradient 1 variance across **A.** control and all TLE subjects, and **B.** control, L-TLE, and R-TLE. **C-E.** 1-dimensional scatter of gradient 1 across all ROIs for 10 subjects (one per line) sorted from most gradient 1 variance (bottom) to least (top) for **C.** control, **D.** L-TLE and **E.** R-TLE.

Previous studies in epilepsy have demonstrated an expansion of the cortical functional gradient 1 in generalized epilepsy relative to controls (de Bézenac et al., 2021). Therefore, we tested whether the subcortical functional gradient was expanded in focal epilepsy relative to controls. We quantified the extent of gradient expansion and contraction by measuring the subject-level variance in the principal gradient across all subcortical ROIs. For R-TLE and L-TLE combined, we found a statistically significant gradient 1 expansion relative to controls (p = 0.0180, one-tailed *t*-test; Cohen’s D = 0.54) (Fig. 4A). For R-TLE and L-TLE separately, we found a statistically significant expansion of gradient 1 in R-TLE relative to controls (p = 0.048, one-tailed *t*-test, Bonferroni corrected; Cohen’s D = 0.68), and a non-statistically significant expansion in L-TLE relative to controls (p = 0.138, one-tailed *t*-test, Bonferroni corrected; Cohen’s D = 0.47) (Fig. 4B). Fig. 4C-E shows representative examples of the spread of gradient 1 across different subjects and different groups. The group level gradient 1 distribution for each group is shown in Supplementary Fig. 5. We also assessed whether further group differences could be captured by additional gradients. No differences were found in gradients’ 2 though 5 global variance, nor across their multivariate dispersion (Bethlehem et al., 2020) in combinations of gradients 1 through 5 (Supplementary Figure 6). These findings suggest a subcortical functional principal gradient (gradient 1) expansion in focal TLE that is similar to that observed in the cortex and thalamus in generalized epilepsy (de Bézenac et al., 2021).

**Fig. 5:**
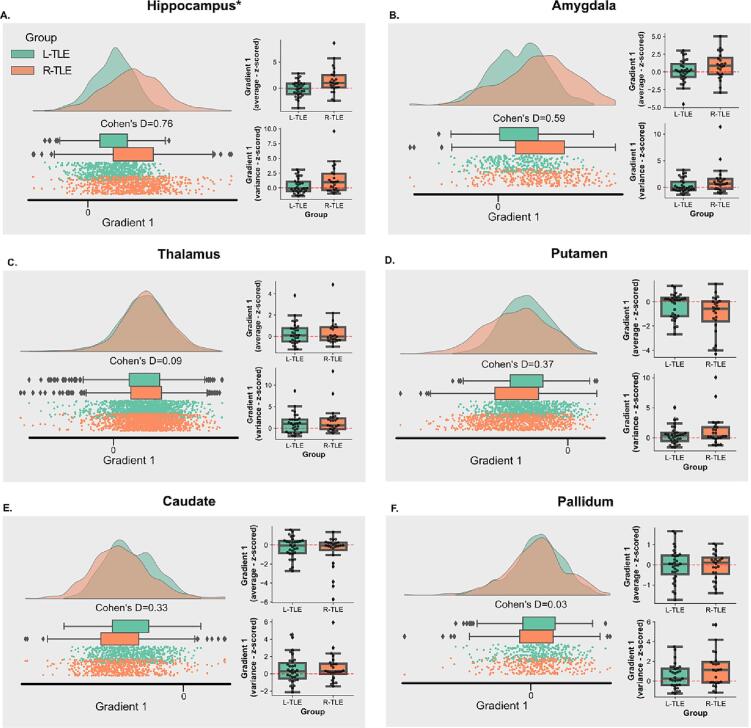
**Principal Gradient 1 Across Ipsilateral Subcortical ROIs: A-F.** Each panel represents a different subcortical ROI, and they show both, the average distribution in gradient space for gradient 1 across subjects in each group (left), and the distribution of individual gradient 1 mean and variance for subjects in each group (right). The individual subject mean and variance were z-scored relative to the distribution of gradient 1 mean and variance for controls in the same ROI, but across bilateral regions.

### 3.3 Hippocampal principal gradient 1 ipsilateral to the SOZ is different in left and right TLE

Given the observed differences in gradient expansion present between left and right TLE, we then explored how the gradient expansion and magnitude differed across subcortical ROIs between left and right TLE. We conducted this analysis both in average gradient space and in subject space using gradient 1 across ROIs ipsilateral to the SOZ. The results are shown in Fig. 5. Average gradient space demonstrates a larger magnitude for the ipsilateral hippocampus in R-TLE relative to L-TLE that trended towards significance (p_BON,PERM_ = 0.063; Cohen’s D = 0.72). At the subject level, the mean gradient 1 value in the ipsilateral hippocampus was also higher for R-TLE than L-TLE (p_BON,PERM_ = 0.032; Cohen’s D = 0.87), with the subject level variance also being larger for R-TLE, but not statistically significant (p_BON,PERM_ = 0.39; Cohen’s D = 0.52). A trend in the same direction was observed in the ipsilateral amygdala: higher group level gradient for R-TLE (p_BON,PERM_ = 0.48; Cohen’s D = 0.59), and higher subject level gradient mean (p_BON,PERM_ = 0.56; Cohen’s D = 0.43) and variance (p_BON,PERM_ = 0.42; Cohen’s D = 0.48), but the findings were not statistically significant. For the thalamus, caudate and pallidum, the differences between L-TLE and R-TLE had small effect sizes (Cohen’s D < 0.50) and no differences were statistically significant (p_BON,PERM_ > 0.05). However, a trend in the opposite direction was observed in the putamen and caudate, with a lower group and subject level mean gradient values in R-TLE relative to L-TLE. These findings suggest that the increased global variance in the principal gradient of R-TLE relative to L-TLE and controls, is driven by more extreme (more positive) principal gradient 1 values in the ipsilateral hippocampus. However, potential contributions by more extreme values in other more computationally complex subcortical areas such as the amygdala, and more extreme values in the opposite direction (more negative values) in less computationally complex subcortical areas, like the putamen and caudate, cannot be fully excluded. No sizeable effects nor significant differences were observed for the contralateral ROIs (Supplementary Figure 7).

Using the Bhattacharyya distance, we wanted to validate whether the findings of the 1st principal gradient in the hippocampus would extend to the group level 2-dimensional distribution generated by principal gradients 1 and 2. We found that the Battacharyya distance was statistically significant between the ipsilateral hippocampus of L-TLE and R-TLE (p_PERM_ = 0.029, Battacharyya distance = 0.10). Distances across other ipsilateral and contralateral ROIs are show in Supplementary Figures 8 and 9.

Overall, these findings demonstrate differences in the ipsilateral hippocampal functional gradient in L-TLE compared to R-TLE, suggesting a cortical connectivity profile in the ipsilateral hippocampus that depends on the laterality of the SOZ.

### 3.4 Clinical variables other than epilepsy laterality had little association with subcortical functional gradients

We used a linear model to assess whether clinical variables, other than disease laterality, influenced the mean and variance of gradient 1. We found that, for the ipsilateral hippocampus, disease laterality remained the only covariate with a statistically significant association with the gradient 1 mean (p = 0.012, corrected across 4 covariates), consistent with the findings in the previous sections of the paper. We also found that the mean gradient 1 of the contralateral caudate had a statistically significant association with the presence of MTS (p = 0.012, corrected across 4 covariates). We additionally found for MTS status, negative coefficients for higher computational regions (hippocampus, amygdala, and thalamus), and positive coefficients for lower computational regions (putamen and caudate). This suggests a trend towards gradient 1 contraction in the presence of MTS, or conversely, an expansion in the absence of MTS. Our data further supports this interpretation with a negative coefficient for MTS in a model predicting the global gradient 1 variance, which demonstrates a decrease in the latter quantity (contraction) in the presence of MTS (Supplementary Table 4). We found no other significant associations between the mean, or variance, of gradient 1 and the presence of mesial temporal sclerosis, history of focal to bilateral tonic-clonic seizures, or disease duration across ROIs (Supplementary Table 2 and 3).

## 4 Discussion

In this study we describe the subcortical-to-cortical functional connectivity signature of temporal lobe epilepsy via the use of functional connectivity gradients. While previous functional gradient studies in epilepsy have largely focused on cortical gradients, here we provide an overview of subcortical-to-cortical functional gradients, which further supports the involvement of these structures in the pathophysiology of temporal lobe epilepsy. Our results demonstrate a lower to higher computational hierarchy in the subcortex that goes from the putamen to the thalamus along principal gradient 1. We also show an expanded subcortical principal gradient in individuals with TLE subjects relative to healthy controls, which is consistent with previous findings in the cortical gradients of patients with generalized epilepsy(de Bézenac et al., 2021), but contradicts previous findings with similar methods in the cortex of patients with TLE(Larivière et al., 2020). Finally, we show that the gradient expansion in TLE was more pronounced in R-TLE than in L-TLE, with this difference being driven by a larger (more positive) principal gradient 1 value in the ipsilateral hippocampus of R-TLE patients.

A contraction of functional connectivity gradients, relative to neurotypical controls, has been previously demonstrated in several neuropsychiatric disorders, including autism spectrum disorder and schizophrenia (Dong et al., 2020, Cook et al., 2019). In epilepsy, however, studies have demonstrated principal cortical gradient expansion in both generalized epilepsy (de Bézenac et al., 2021), and more recently, in newly diagnosed focal epilepsy (Haneef et al., 2012). Our findings add to the growing body of evidence of an expanded functional gradient in epilepsy, by demonstrating that functional gradient expansion also occurs in the subcortex of patients with TLE. These findings in combination suggest that principal gradient expansion is a characteristic feature of the functional connectome of epilepsy both at the cortical level as well as within the subcortex.

An expanded subcortical functional gradient is evidence of a connectivity profile within subcortical voxels that is more variable in TLE than it is healthy controls. Broadly, this means that two voxels that have a similar connectivity pattern in healthy controls and, therefore, are close together in diffusion embedding space in these, are less likely to have a similar connectivity pattern in individuals with epilepsy, therefore they will be further apart in diffusion embedding space. Furthermore, our findings suggest that the increased variation in connectivity is driven by changes in the hippocampus ipsilateral to the SOZ. Mechanistically, this is consistent with current evidence on TLE, in that we would expect a disrupted connectivity pattern in the hippocampus based on prior structural and functional MRI studies (James et al., 2013, Das et al., 2009, Alves et al., 2019, Chiang et al., 2014). Additionally, some research avenues place the hippocampus as a node within the default mode network (He et al., 2020), which is where changes have been previously demonstrated to drive gradient expansion in epilepsy (de Bézenac et al., 2021). Our findings therefore are consistent and complementary to current neuroimaging research in epilepsy.

Differences between left and right TLE have been demonstrated in the past both in functional and structural neuroimaging studies, and while there is not universal agreement in the literature, many studies report that right TLE has either stronger, or more widespread, abnormalities (Lemkaddem et al., 2014, Voets et al., 2012, Voets et al., 2011, Trenerry et al., 1993, Park et al., 2018). These reported functional and structural alterations in R-TLE are consistent with the findings of this study, since a stronger expansion of the principal gradient in R-TLE could represent a more widely distributed subcortical, and more specifically, hippocampal, connectivity network. Our findings contribute to the hypothesis of left and right TLE as two distinct epilepsy phenotypes that differ on more than the laterality of the disease, with widespread hippocampo-cortical connectivity abnormalities as part of this phenotype.

In this study, we found that disease laterality was the main factor that seemed to influence gradient values. We also found an association between the contralateral caudate and MTS, which at a larger scale, represented a trend towards global gradient contraction in the presence of MTS, or consequently, an expansion in non-lesional TLE. This might be evidence of a more distributed epileptic network in non-lesional TLE, but our results are preliminary, and further confirmation is needed. As for other clinical factors, history of focal to bilateral tonic-clonic seizures and disease duration were not related to gradient values. There are several reasons as to why this might be the case. First, it is possible that during the dimensionality reduction implemented during gradient generation, the variance of disease laterality dominates over the variance of the other covariates, causing the gradient representation to encode differences in disease laterality much better than differences in other disease factors. Second, it is possible that with more granular ROIs we could obtain more specific gradient changes due to disease factors. For example, quantifying the gradient properties within thalamic subnuclei, such as the mediodorsal nucleus, we might be able to capture differences between patients with and without a history of BTCS (Bonilha et al., 2006). Third, it is possible that the temporal signal-to-noise ratio (tSNR) of the BOLD signal in subcortical structures measured at 3 T is not sufficient to discriminate nuances in disease factors at a gradient level, making a higher field strength (e.g. 7 T) functional acquisition a more appropriate approach for these questions. Finally, it is also possible that the functional signature of subcortical structures is not sensitive to these disease factors and is instead inherent to the disease itself. This can be particularly true for disease duration, where even in clear structural abnormalities like hippocampal sclerosis, it is still controversial whether disease duration has an impact on hippocampal volume (Dale et al., 1999, Yushkevich et al., 2015, Su et al., 2019).

Our study has several limitations. First, our sample size is moderate. Future multicenter epilepsy studies should attempt to validate our findings using larger sample sizes. Second, gradient representations provide a global characterization of the connectivity from a subcortical voxel to the cortex, however, there is no established methodology for identifying the cortical regions that are driving the subcortical gradient differences. Future work in the functional gradient literature should focus on developing efficient strategies for mapping the gradient differences back to target cortical voxels. This would allow researchers in the field to confirm whether the differences between R and L-TLE that we reported in the ipsilateral hippocampus are driven by connectivity differences in the default mode network, or other cortical regions. Finally, we made use of subcortical ROIs that were defined a priori through a standard atlas. While appropriate for this exploratory study, future studies should leverage subject-specific subcortical segmentations, such as those provided by FreeSurfer[50], ASHS[51] and THOMAS[52]. Additionally, usage of ultra-high field imaging at 7 T can not only help get more accurate segmentations of these structures, but also improved tSNR in the BOLD signal, which would precisely localize signals within the subcortex, further elucidating differences between epilepsy subtypes and disease factors in gradient space.

## 5 Conclusions

In this study we describe the subcortical-to-cortical functional connectivity signature of temporal lobe epilepsy through functional connectivity gradients, demonstrating an expansion of the principal subcortical gradient in individuals with epilepsy relative to healthy controls. These findings indicate gradient expansion as a functional connectome phenotype in epilepsy. We also demonstrate differences in the gradient between L-TLE and R-TLE that may be driven by changes in the hippocampus ipsilateral to the seizure onset zone.

## 6 Data availability statement

The processed data and methodological pipeline used in this study are available on GitHub: https://github.com/penn-cnt/TLE_Gradients.

## 7 Guidelines on research ethics and publishing

We confirm that we have read the Journal’s position on issues involved in ethical publication and affirm that this report is consistent with those guidelines.

## Funding

AL and KAD received support from NINDS (R01NS116504). NS received support from American Epilepsy Society (953257) and NINDS (R01NS116504). The authors would also like to acknowledge the Thornton Foundation for its generous support.

## CRediT authorship contribution statement

**Alfredo Lucas:** Conceptualization, Methodology, Software, Validation, Formal analysis, Writing – original draft. **Sofia Mouchtaris:** Conceptualization, Methodology, Software, Validation, Formal analysis, Writing – original draft. **Eli J. Cornblath:** Conceptualization, Writing – review & editing, Data curation. **Nishant Sinha:** Conceptualization, Writing – review & editing. **Lorenzo Caciagli:** Conceptualization, Writing – review & editing. **Peter Hadar:** Data curation, Writing – review & editing. **James J. Gugger:** Conceptualization, Writing – review & editing. **Sandhitsu Das:** Data curation, Writing – review & editing. **Joel M. Stein:** Data curation, Writing – review & editing. **Kathryn A. Davis:** Supervision, Project administration, Funding acquisition, Data curation, Conceptualization, Writing – review & editing.

## Declaration of Competing Interest

The authors declare that they have no known competing financial interests or personal relationships that could have appeared to influence the work reported in this paper.

## Data Availability

The processed data and methodological pipeline used in this study are available on GitHub: https://github.com/penn-cnt/TLE_Gradients

## References

[b0005] Alves P.N., Foulon C., Karolis V., Bzdok D., Margulies D.S., Volle E., Thiebaut de Schotten M. (2019). An improved neuroanatomical model of the default-mode network reconciles previous neuroimaging and neuropathological findings. Commun. Biol..

[b0010] Bassett D.S., Bullmore E. (2006). Small-world brain networks. Neuroscientist.

[b0015] Bernhardt B.C., Smallwood J., Keilholz S., Margulies D.S. (2022). Gradients in brain organization. Neuroimage.

[b0020] Bethlehem R.A.I., Paquola C., Seidlitz J., Ronan L., Bernhardt B., Consortium C.-CAN., Tsvetanov K.A. (2020). Dispersion of functional gradients across the adult lifespan. Neuroimage.

[b0025] Bonilha L., Rorden C., Appenzeller S., Carolina Coan A., Cendes F., Min L.L. (2006). Gray matter atrophy associated with duration of temporal lobe epilepsy. Neuroimage.

[b0030] Bullmore E., Sporns O. (2009). Complex brain networks: graph theoretical analysis of structural and functional systems. Nat. Rev. Neurosci..

[b0035] Caciagli L., Bernhardt B.C., Hong S.J., Bernasconi A., Bernasconi N. (2014). Functional network alterations and their structural substrate in drug-resistant epilepsy. Front. Neurosci..

[b0040] Caciagli L, Paquola C, He X, Vollmar C, Centeno M, Wandschneider B, Braun U, Trimmel K, Vos SB, Sidhu MK, Thompson PJ, Baxendale S, Winston GP, Duncan JS, Bassett DS, Koepp MJ, Bernhardt BC. Disorganization of language and working memory systems in frontal versus temporal lobe epilepsy. *Brain*. 10.1093/brain/awac150 PMC9976988 35511160

[b0045] Chiang S., Stern J.M., Engel J., Levin H.S., Haneef Z. (2014). Differences in graph theory functional connectivity in left and right temporal lobe epilepsy. Epilepsy Res..

[b0050] Ćirić R, Azeez Adebimpe, Cieslak M, et al. PennBBL/xcpEngine: compatible with fmriprep outputs of version 20.07. Published online May 23, 2020. doi:10.5281/ZENODO.3840960.

[b0055] Cook C.J., Hwang G., Mathis J., Nair V.A., Conant L.L., Allen L., Almane D.N., Birn R., DeYoe E.A., Felton E., Forseth C., Humphries C.J., Kraegel P., Nencka A., Nwoke O., Raghavan M., Rivera-Bonet C., Rozman M., Tellapragada N., Ustine C., Ward B.D., Struck A., Maganti R., Hermann B., Prabhakaran V., Binder J.R., Meyerand M.E. (2019). Effective connectivity within the default mode network in left temporal lobe epilepsy: Findings from the epilepsy connectome project. Brain Connect..

[b0060] Dale A.M., Fischl B., Sereno M.I. (1999). Cortical surface-based analysis I. Segmentation and surface reconstruction. NeuroImage..

[b0065] Das S.R., Mechanic-Hamilton D., Korczykowski M. (2009). Structure specific analysis of the hippocampus in temporal lobe epilepsy. Hippocampus.

[b0070] Bézenac CE de, Caciagli L, Alonazi BK, Bernhardt BC, Marson AG, Keller SS. Altered functional connectome hierarchy with gene expression signatures in newly-diagnosed focal epilepsy. *medRxiv*. Published online 2021:2021.07.18.21259977. doi:10.1101/2021.07.18.21259977.

[b0075] Dong D., Luo C., Guell X., Wang Y., He H., Duan M., Eickhoff S.B., Yao D. (2020). Compression of cerebellar functional gradients in schizophrenia. Schizophr. Bull..

[b0080] Esteban O., Markiewicz C.J., Blair R.W., Moodie C.A., Isik A.I., Erramuzpe A., Kent J.D., Goncalves M., DuPre E., Snyder M., Oya H., Ghosh S.S., Wright J., Durnez J., Poldrack R.A., Gorgolewski K.J. (2019). fMRIPrep: a robust preprocessing pipeline for functional MRI. Nat. Methods.

[b0085] Fadaie F., Lee H.M., Caldairou B., Gill R.S., Sziklas V., Crane J., Bernhardt B.C., Hong S.-J., Bernasconi A., Bernasconi N. (2021). Atypical functional connectome hierarchy impacts cognition in temporal lobe epilepsy. Epilepsia.

[b0090] Fortin J.-P., Cullen N., Sheline Y.I., Taylor W.D., Aselcioglu I., Cook P.A., Adams P., Cooper C., Fava M., McGrath P.J., McInnis M., Phillips M.L., Trivedi M.H., Weissman M.M., Shinohara R.T. (2018). Harmonization of cortical thickness measurements across scanners and sites. Neuroimage.

[b0095] Haak K.V., Marquand A.F., Beckmann C.F. (2018). Connectopic mapping with resting-state fMRI. Neuroimage.

[b0100] Haneef Z., Lenartowicz A., Yeh H.J., Engel J., Stern J.M. (2012). Effect of lateralized temporal lobe epilepsy on the default mode network. Epilepsy Behav..

[b0105] He X., Chaitanya G., Asma B., Caciagli L., Bassett D.S., Tracy J.I., Sperling M.R. (2020). Disrupted basal ganglia–thalamocortical loops in focal to bilateral tonic-clonic seizures. Brain.

[b0110] Hong S.-J., Vos de Wael R., Bethlehem R.A.I., Lariviere S., Paquola C., Valk S.L., Milham M.P., Di Martino A., Margulies D.S., Smallwood J., Bernhardt B.C. (2019). Atypical functional connectome hierarchy in autism. Nat. Commun..

[b0115] Hong S.-J., Xu T., Nikolaidis A., Smallwood J., Margulies D.S., Bernhardt B., Vogelstein J., Milham M.P. (2020). Toward a connectivity gradient-based framework for reproducible biomarker discovery. Neuroimage.

[b0120] James G.A., Tripathi S.P., Ojemann J.G., Gross R.E., Drane D.L. (2013). Diminished default mode network recruitment of the hippocampus and parahippocampus in temporal lobe epilepsy: Clinical article. J. Neurosurg..

[b0125] Kramer M.A., Cash S.S. (2012). Epilepsy as a disorder of cortical network organization. Neuroscientist.

[b0130] Kwan P., Brodie M.J. (2000). Early identification of refractory epilepsy. N. Engl. J. Med..

[b0135] Larivière S., Weng Y., Vos de Wael R., Royer J., Frauscher B., Wang Z., Bernasconi A., Bernasconi N., Schrader D.V., Zhang Z., Bernhardt B.C. (2020). Functional connectome contractions in temporal lobe epilepsy: Microstructural underpinnings and predictors of surgical outcome. Epilepsia.

[b0140] Lee M.H., Smyser C.D., Shimony J.S. (2013). Resting-state fMRI: A review of methods and clinical applications. Am. J. Neuroradiol..

[b0145] Lemkaddem A., Daducci A., Kunz N., Lazeyras F., Seeck M., Thiran J.-P., Vulliémoz S. (2014). Connectivity and tissue microstructural alterations in right and left temporal lobe epilepsy revealed by diffusion spectrum imaging. NeuroImage Clin..

[b0150] Lucas A., Cornblath E.J., Sinha N., Hadar P., Caciagli L., Keller S.S., Bonilha L., Shinohara R.T., Stein J.M., Das S., Gleichgerrcht E., Davis K.A. (2023 May). Resting state functional connectivity demonstrates increased segregation in bilateral temporal lobe epilepsy. Epilepsia..

[b0155] Margulies D.S., Ghosh S.S., Goulas A., Falkiewicz M., Huntenburg J.M., Langs G., Bezgin G., Eickhoff S.B., Castellanos F.X., Petrides M., Jefferies E., Smallwood J. (2016). Situating the default-mode network along a principal gradient of macroscale cortical organization. Proc. Natl. Acad. Sci..

[b0160] Meng Y., Yang S., Chen H., Li J., Xu Q., Zhang Q., Lu G., Zhang Z., Liao W. (2021). Systematically disrupted functional gradient of the cortical connectome in generalized epilepsy: Initial discovery and independent sample replication. Neuroimage.

[b0165] B.-Y. Park S.-J. Hong S.L. Valk C. Paquola O. Benkarim R.A.I. Bethlehem A. Di Martino M.P. Milham A. Gozzi B.T.T. Yeo J. Smallwood B.C. Bernhardt Differences in subcortico-cortical interactions identified from connectome and microcircuit models in autism Nat Commun. 12 1 2021;12(1):2225. 10.1038/s41467-021-21732-0. 10.1038/s41467-021-21732-0 PMC8044226 33850128

[b0170] Park K.M., Kim T.H., Mun C.W., Shin K.J., Ha S.Y., Park JinSe, Lee B.I., Lee H.-J., Kim S.E. (2018). Reduction of ipsilateral thalamic volume in temporal lobe epilepsy with hippocampal sclerosis. J. Clin. Neurosci..

[b0175] Pedersen M., Omidvarnia A.H., Walz J.M., Jackson G.D. (2015). Increased segregation of brain networks in focal epilepsy: An fMRI graph theory finding. NeuroImage Clin..

[b0180] De la Porte J., Herbst B., Hereman W., van der Walt S. (2008, November). An Introduction to diffusion maps. In Proceedings of the 19th symposium of the pattern recognition association of South Africa (PRASA 2008), Cape Town, South Africa.

[b0185] Querol Pascual M.R. (2007). Temporal lobe epilepsy: clinical semiology and neurophysiological studies. Semin Ultrasound CT MRI..

[b0190] Schönemann P.H. (1966). A generalized solution of the orthogonal procrustes problem. Psychometrika.

[b0195] Su J.H., Thomas F.T., Kasoff W.S., Tourdias T., Choi E.Y., Rutt B.K., Saranathan M. (2019). Thalamus optimized multi atlas segmentation (THOMAS): fast, fully automated segmentation of thalamic nuclei from structural MRI. Neuroimage.

[b0200] Tian Y., Margulies D.S., Breakspear M., Zalesky A. (2020). Topographic organization of the human subcortex unveiled with functional connectivity gradients. Nat. Neurosci..

[b0205] Trenerry M.R., Jack C.R., Sharbrough F.W., Cascino G.D., Hirschorn K.A., Marsh W.R., Kelly P.J., Meyer F.B. (1993). Quantitative MRI hippocampal volumes: association with onset and duration of epilepsy, and febrile convulsions in temporal lobectomy patients. Epilepsy Res..

[b0210] Trimmel K., Vos S.B., Caciagli L., Xiao F., Graan L.A., Winston G.P., Koepp M.J., Thompson P.J., Duncan J.S. (2021). Decoupling of functional and structural language networks in temporal lobe epilepsy. Epilepsia.

[b0215] Voets N.L., Bernhardt B.C., Kim H., Yoon U., Bernasconi N. (2011). Increased temporolimbic cortical folding complexity in temporal lobe epilepsy. Neurology.

[b0220] Voets N.L., Beckmann C.F., Cole D.M., Hong S., Bernasconi A., Bernasconi N. (2012). Structural substrates for resting network disruption in temporal lobe epilepsy. Brain.

[b0225] R. Vos de Wael O. Benkarim C. Paquola S. Lariviere J. Royer S. Tavakol T. Xu S.-J. Hong G. Langs S. Valk B. Misic M. Milham D. Margulies J. Smallwood B.C. Bernhardt BrainSpace: a toolbox for the analysis of macroscale gradients in neuroimaging and connectomics datasets Commun Biol. 3 1 2020;3(1):103. 10.1038/s42003-020-0794-7. 10.1038/s42003-020-0794-7 PMC7058611 32139786

[b0230] Xiao F., An D., Zhou D. (2017). Functional MRI-based connectivity analysis: A promising tool for the investigation of the pathophysiology and comorbidity of epilepsy. Seizure.

[b0235] Yang S., Meng Y., Li J., Li B., Fan Y.-S., Chen H., Liao W. (2020). The thalamic functional gradient and its relationship to structural basis and cognitive relevance. Neuroimage.

[b0240] Yushkevich P.A., Pluta J.B., Wang H., Xie L., Ding S.-L., Gertje E.C., Mancuso L., Kliot D., Das S.R., Wolk D.A. (2015). Automated volumetry and regional thickness analysis of hippocampal subfields and medial temporal cortical structures in mild cognitive impairment. Hum. Brain Mapp..

[b0245] Zhang Z., Lu G., Zhong Y., Tan Q., Liao W., Wang Z., Wang Z., Li K., Chen H., Liu Y. (2010). Altered spontaneous neuronal activity of the default-mode network in mesial temporal lobe epilepsy. Brain Res..

