# Supplementary material for: Subcortical functional connectivity gradients in temporal lobe epilepsy
Source: Neuroimage Clin. 2023 May 5;38:103418. doi: 10.1016/j.nicl.2023.103418 (PMC10196948; doi:10.1016/j.nicl.2023.103418)
Supplement: Supplementary data 1 [file mmc1.docx]

**A Description of Subcortical Functional Connectivity Gradients in Temporal Lobe Epilepsy**

Supplementary Figures and Tables

**Supplementary Table 1 –** Individual subject clinical characteristics and demographics. **Abbreviations:** L – Left. R – Right. B – Bilateral. EMU – Epilepsy monitoring unit. BTCS – History of bilateral tonic clonic seizures. MTS – Mesial temporal sclerosis. TBI – Traumatic brain injury. PET – Positron emission tomography. FCD – Focal cortical dysplasia.

| **Subject** | **Age** | **Sex** | **Intracranial EEG** | **Age at Onset** | **BTCS** | **MRI Lesional** | **MRI Lesion Laterality** | **Presence of MTS** | **Suspected Etiology** | **Etiology Details** | **PET Lateralization (hypometabolism)** | **Final Surgical Conference Lateralization** | **Engel Outcome** | **Note** |
| --- | --- | --- | --- | --- | --- | --- | --- | --- | --- | --- | --- | --- | --- | --- |
| 1 | 46.1 | F | TRUE | 6 | Yes | Nonlesional |  | No | Idiopathic |  | R | R | IID |  |
| 2 | 49 | F | FALSE | 5 | Yes | Lesional | R | Yes | MTS |  | R | R |  |  |
| 3 | 24.8 | M | TRUE | 4 | Yes | Nonlesional |  | No | Idiopathic |  | R | R | IB |  |
| 4 | 31 | M | FALSE | 30 | Yes | Nonlesional |  | No | Idiopathic |  | R | R |  |  |
| 5 | 47.3 | F | TRUE | 26 | Yes | Lesional | L | Yes | MTS |  | L | L | IB |  |
| 6 | 54.8 | M | TRUE | 28 | No | Lesional | B | No | Structural | Subependymal heterotopia | L | L |  |  |
| 7 | 48 | F | FALSE | 2 | Yes | Lesional | R | Yes | MTS |  | R | R |  |  |
| 8 | 26.8 | M | TRUE | 12 | Yes | Nonlesional |  | No | Idiopathic |  | L | L |  |  |
| 9 | 35.3 | F | FALSE | 1 | Yes | Lesional | L | No | TBI |  | L | L |  |  |
| 10 | 30 | M | FALSE | 13 | Yes | Lesional | R | Yes | MTS |  | R | R |  |  |
| 11 | 27.8 | F | TRUE | 2 | Yes | Nonlesional |  | No | Idiopathic |  | R | L |  | Left onset confirmed with iEEG |
| 12 | 36 | M | TRUE | 5 | No | Nonlesional |  | No | Idiopathic |  | R | R | IA |  |
| 13 | 48.1 | F | FALSE | 41 | Yes | Lesional | R | Yes | MTS |  | R | R |  |  |
| 14 | 29.1 | M | TRUE | 28 | No | Lesional | L | No | TBI | Encephalomalacia | L | L |  |  |
| 15 | 35 | M | TRUE | 25 | Yes | Lesional | B | No | TBI | Bilateral Frontal, R temporal encephalomalacia | B | L |  | Left onset confirmed with iEEG |
| 16 | 67.2 | M | FALSE | 37 | No | Lesional | R | No | Stroke | Old hemorrhage, encephalomalacia | R | R |  |  |
| 17 | 25.8 | M | TRUE | 25 | Yes | Lesional | L | Yes | MTS |  | L | L | IB |  |
| 18 | 45.2 | F | TRUE | 24 | Yes | Lesional | L | No | Structural | Polymicrogyria | L | R |  | Right onset confirmed with iEEG |
| 19 | 40.8 | F | FALSE | 0.333333333 | Yes | Nonlesional |  | No | Idiopathic |  | None | L |  | Lateralization determined based on clinical semiology and EMU scalp EEG |
| 20 | 60.1 | F | FALSE | 42 | No | Nonlesional |  | No | Idiopathic |  | R | R |  |  |
| 21 | 46.7 | M | FALSE | 35 | No | Lesional | R | Yes | MTS |  | R | R |  |  |
| 22 | 55 | M | FALSE | 15 | Yes | Lesional | L | Yes | MTS |  | L | L | IA |  |
| 23 | 48.1 | F | FALSE | 7 | Yes | Lesional | L | Yes | MTS |  | L | L |  |  |
| 24 | 22.2 | M | TRUE | 19 | Yes | Lesional | L | No | Infection | Meningoencephalitis | L | L | IA |  |
| 25 | 29.2 | M | FALSE | 18 | Yes |  |  | No | Structural | FCD | L | L | IIA |  |
| 26 | 24.1 | M | TRUE | 19 | Yes | Nonlesional |  | No | Idiopathic |  | R | R |  |  |
| 27 | 49.7 | F | FALSE | 32 | Yes | Lesional | L | No | Infection | Meningitis | L | L | IA |  |
| 28 | 27 | M | TRUE | 16 | Yes | Nonlesional |  | No | Idiopathic |  | L | L | IIIA |  |
| 29 | 39.7 | F | TRUE | 37 | No | Nonlesional | L | No | Idiopathic |  | L | L |  |  |
| 30 | 52.7 | M | FALSE | 5 | Yes | Nonlesional | L | No | Idiopathic |  | L | L |  |  |
| 31 | 35 | M | FALSE | 29 | No | Lesional | L | No | Structural | DNET v glioma | L | L | IB |  |
| 32 | 21.8 | F | FALSE | 2 | No | Nonlesional |  | No | Idiopathic |  | L | L | IA |  |
| 33 | 35 | F | TRUE | 15 | No | Lesional | L | No | Structural | Encephalocele | L | L | IIIA |  |
| 34 | 36.7 | F | FALSE | 26 | Yes | Lesional | R | Yes | MTS |  | R | R |  |  |
| 35 | 28 | F | FALSE | 23 | Yes | Nonlesional |  | No | Idiopathic |  | L | L |  |  |
| 36 | 19.7 | F | TRUE | 13 | Yes | Lesional | L | No | Stroke | L BG Infarct | L | L |  |  |
| 37 | 50.4 | F | FALSE | 5 | No | Lesional | R | No | Infection | Meningitis | R | R | IA |  |
| 38 | 49.4 | F | TRUE | 28 | No | Nonlesional |  | No | Idiopathic |  | L | L |  |  |
| 39 | 35.3 | M | FALSE | 35 | Yes | Nonlesional |  | No | Idiopathic |  | R | R |  |  |
| 40 | 39 | M | FALSE | 35 | Yes | Lesional | R | No | Structural | MTS | R | R |  |  |
| 41 | 59 | F | FALSE | 56 | No | Lesional | R | No | Structural; Surgery | R temporal Encephalocele | R | R |  |  |
| 42 | 30.7 | F | TRUE | 16 | Yes | Lesional | B | No | Structural | R cingulate dysplasia; L MTS | L | L | IIB |  |
| 43 | 27 | F | FALSE | 20 | No | Lesional | L | Yes | MTS |  | L | L |  |  |
| 44 | 32.9 | M | TRUE | 19 | Yes | Nonlesional |  | No | Idiopathic |  | L | R |  | Right onset confirmed with iEEG |
| 45 | 18.9 | M | FALSE | 7 | Yes | Lesional | L | No | Structural | Encephalomalacia | L | L |  |  |
| 46 | 36.6 | F | TRUE | 16 | Yes | Nonlesional |  | No | Idiopathic |  | L | L | IIB |  |
| 47 | 33.4 | M | FALSE | 30 | Yes | Lesional | L | No | Structural | Encephalocele L temporal | L | L |  |  |
| 48 | 19.9 | F | TRUE | 12 | No | Lesional | R | Yes | MTS |  | R | R | IIB |  |
| 49 | 39 | F | TRUE | 15 | Yes | Lesional | R | No | Structural | DVA | R | R | IIB |  |
| 50 | 20.1 | F | TRUE | 18 | No | Lesional | R | Yes | Structural | MTS | R | R | IB |  |
| 51 | 29 | M | TRUE | 27 | Yes | Lesional | L | No | Structural | (1) left posterior insular cortical dysplasia abutting the superior temporal gyrus (2) left anterior temporal meningocele (3) left lateral temporal meningocele (4) left anterior sub-insular lesion favored to be VR spaces much more likely than DNET) | L | L | IB |  |
| 52 | 35.9 | M | FALSE | 33 | Yes | Lesional | R | No | Surgery | Mass resection, Right uncal signal | R | R |  |  |
| 53 | 34.8 | M | TRUE | 30 | Yes | Lesional | L | No | Structural | L MTG FCD | None | L |  |  |
| 54 | 24 | F | FALSE | 3 | Yes | Lesional | L | Yes | MTS |  | L | L |  |  |
| 55 | 25.1 | M | FALSE | 19 | Yes | Lesional | L | Yes | MTS |  | L | L |  |  |

| **Subject** | **Age** | **Sex** | **Intracranial EEG** | **Age at Onset** | **BTCS** | **MRI Lesional** | **MRI Lesion Laterality** | **Presence of MTS** | **Suspected Etiology** | **Etiology Details** | **PET Lateralization (hypometabolism)** | **Final Surgical Conference Lateralization** | **Engel Outcome** | **Note** |
| --- | --- | --- | --- | --- | --- | --- | --- | --- | --- | --- | --- | --- | --- | --- |
| 20 | 60.1 | F | FALSE | 42 | No | Nonlesional |  | No | Idiopathic |  | R | R |  |  |
| 21 | 46.7 | M | FALSE | 35 | No | Lesional | R | Yes | MTS |  | R | R |  |  |
| 22 | 55 | M | FALSE | 15 | Yes | Lesional | L | Yes | MTS |  | L | L | IA |  |
| 23 | 48.1 | F | FALSE | 7 | Yes | Lesional | L | Yes | MTS |  | L | L |  |  |
| 24 | 22.2 | M | TRUE | 19 | Yes | Lesional | L | No | Infection | Meningoencephalitis | L | L | IA |  |
| 25 | 29.2 | M | FALSE | 18 | Yes | Lesional | L | No | Structural | FCD | L | L | IIA |  |
| 26 | 24.1 | M | TRUE | 19 | Yes | Nonlesional |  | No | Idiopathic |  | R | R |  |  |
| 27 | 49.7 | F | FALSE | 32 | Yes | Lesional | L | No | Infection | Meningitis | L | L | IA |  |
| 28 | 27 | M | TRUE | 16 | Yes | Nonlesional |  | No | Idiopathic |  | L | L | IIIA |  |
| 29 | 39.7 | F | TRUE | 37 | No | Nonlesional | L | No | Idiopathic |  | L | L |  |  |
| 30 | 52.7 | M | FALSE | 5 | Yes | Nonlesional | L | No | Idiopathic |  | L | L |  |  |
| 31 | 35 | M | FALSE | 29 | No | Lesional | L | No | Structural | Tumor | L | L | IB |  |
| 32 | 21.8 | F | FALSE | 2 | No | Nonlesional |  | No | Idiopathic |  | L | L | IA |  |
| 33 | 35 | F | TRUE | 15 | No | Lesional | L | No | Structural | Encephalocele | L | L | IIIA |  |
| 34 | 36.7 | F | FALSE | 26 | Yes | Lesional | R | Yes | MTS |  | R | R |  |  |
| 35 | 28 | F | FALSE | 23 | Yes | Nonlesional |  | No | Idiopathic |  | L | L |  |  |
| 36 | 19.7 | F | TRUE | 13 | Yes | Lesional | L | No | Stroke | basal ganglia Infarct | L | L |  |  |
| 37 | 50.4 | F | FALSE | 5 | No | Lesional | R | No | Infection | Meningitis | R | R | IA |  |
| 38 | 49.4 | F | TRUE | 28 | No | Nonlesional |  | No | Idiopathic |  | L | L |  |  |
| 39 | 35.3 | M | FALSE | 35 | Yes | Nonlesional |  | No | Idiopathic |  | R | R |  |  |
| 40 | 39 | M | FALSE | 35 | Yes | Lesional | R | No | Structural | MTS | R | R |  |  |
| 41 | 59 | F | FALSE | 56 | No | Lesional | R | No | Structural; Surgery | R temporal Encephalocele | R | R |  |  |
| 42 | 30.7 | F | TRUE | 16 | Yes | Lesional | B | No | Structural | R cingulate dysplasia; L MTS | L | L | IIB |  |
| 43 | 27 | F | FALSE | 20 | No | Lesional | L | Yes | MTS |  | L | L |  |  |
| 44 | 32.9 | M | TRUE | 19 | Yes | Nonlesional |  | No | Idiopathic |  | L | R |  | Right onset confirmed with iEEG |
| 45 | 18.9 | M | FALSE | 7 | Yes | Lesional | L | No | Structural | Encephalomalacia | L | L |  |  |
| 46 | 36.6 | F | TRUE | 16 | Yes | Nonlesional |  | No | Idiopathic |  | L | L | IIB |  |
| 47 | 33.4 | M | FALSE | 30 | Yes | Lesional | L | No | Structural | Encephalocele L temporal | L | L |  |  |
| 48 | 19.9 | F | TRUE | 12 | No | Lesional | R | Yes | MTS |  | R | R | IIB |  |
| 49 | 39 | F | TRUE | 15 | Yes | Lesional | R | No | Structural | DVA | R | R | IIB |  |
| 50 | 20.1 | F | TRUE | 18 | No | Lesional | R | Yes | Structural | MTS | R | R | IB |  |
| 51 | 29 | M | TRUE | 27 | Yes | Lesional | L | No | Structural | (1) left posterior insular cortical dysplasia abutting the superior temporal gyrus (2) left anterior temporal meningocele (3) left lateral temporal meningocele (4) left anterior sub-insular lesion favored to be VR spaces much more likely than DNET) | L | L | IB |  |
| 52 | 35.9 | M | FALSE | 33 | Yes | Lesional | R | No | Surgery | Mass resection, Right uncal signal | R | R |  |  |
| 53 | 34.8 | M | TRUE | 30 | Yes | Lesional | L | No | Structural | L MTG FCD | None | L |  |  |
| 54 | 24 | F | FALSE | 3 | Yes | Lesional | L | Yes | MTS |  | L | L |  |  |
| 55 | 25.1 | M | FALSE | 19 | Yes | Lesional | L | Yes | MTS |  | L | L |  |  |

| **Subject** | **Age** | **Sex** | **Intracranial EEG** | **Age at Onset** | **BTCS** | **MRI Lesional** | **MRI Lesion Laterality** | **Presence of MTS** | **Suspected Etiology** | **Etiology Details** | **PET Lateralization (hypometabolism)** | **Final Surgical Conference Lateralization** | **Engel Outcome** | **Note** |
| --- | --- | --- | --- | --- | --- | --- | --- | --- | --- | --- | --- | --- | --- | --- |
| 48 | 19.9 | F | TRUE | 12 | No | Lesional | R | Yes | MTS |  | R | R | IIB |  |
| 49 | 39 | F | TRUE | 15 | Yes | Lesional | R | No | Structural | Developmental venous anomaly | R | R | IIB |  |
| 50 | 20.1 | F | TRUE | 18 | No | Lesional | R | Yes | Structural | MTS | R | R | IB |  |
| 51 | 29 | M | TRUE | 27 | Yes | Lesional | L | No | Structural | left posterior insular cortical dysplasia abutting the superior temporal gyrus | L | L | IB |  |
| 52 | 35.9 | M | FALSE | 33 | Yes | Lesional | R | No | Surgery |  | R | R |  |  |
| 53 | 34.8 | M | TRUE | 30 | Yes | Lesional | L | No | Structural | L mesial temporal gyrus FCD | None | L |  |  |
| 54 | 24 | F | FALSE | 3 | Yes | Lesional | R | Yes | MTS |  | R | R |  |  |
| 55 | 25.1 | M | FALSE | 19 | Yes | Lesional | L | Yes | MTS |  | L | L |  |  |
|  |  |  |  |  |  |  |  |  |  |  |  |  |  |  |
|  |  |  |  |  |  |  |  |  |  |  |  |  |  |  |
|  |  |  |  |  |  |  |  |  |  |  |  |  |  |  |
|  |  |  |  |  |  |  |  |  |  |  |  |  |  |  |
|  |  |  |  |  |  |  |  |  |  |  |  |  |  |  |
|  |  |  |  |  |  |  |  |  |  |  |  |  |  |  |
|  |  |  |  |  |  |  |  |  |  |  |  |  |  |  |
|  |  |  |  |  |  |  |  |  |  |  |  |  |  |  |
|  |  |  |  |  |  |  |  |  |  |  |  |  |  |  |
|  |  |  |  |  |  |  |  |  |  |  |  |  |  |  |
|  |  |  |  |  |  |  |  |  |  |  |  |  |  |  |
|  |  |  |  |  |  |  |  |  |  |  |  |  |  |  |
|  |  |  |  |  |  |  |  |  |  |  |  |  |  |  |
|  |  |  |  |  |  |  |  |  |  |  |  |  |  |  |
|  |  |  |  |  |  |  |  |  |  |  |  |  |  |  |
|  |  |  |  |  |  |  |  |  |  |  |  |  |  |  |
|  |  |  |  |  |  |  |  |  |  |  |  |  |  |  |
|  |  |  |  |  |  |  |  |  |  |  |  |  |  |  |
|  |  |  |  |  |  |  |  |  |  |  |  |  |  |  |
|  |  |  |  |  |  |  |  |  |  |  |  |  |  |  |
|  |  |  |  |  |  |  |  |  |  |  |  |  |  |  |
|  |  |  |  |  |  |  |  |  |  |  |  |  |  |  |
|  |  |  |  |  |  |  |  |  |  |  |  |  |  |  |
|  |  |  |  |  |  |  |  |  |  |  |  |  |  |  |
|  |  |  |  |  |  |  |  |  |  |  |  |  |  |  |
|  |  |  |  |  |  |  |  |  |  |  |  |  |  |  |
|  |  |  |  |  |  |  |  |  |  |  |  |  |  |  |
|  |  |  |  |  |  |  |  |  |  |  |  |  |  |  |

**Supplementary Table 2 –** Regression coefficients and corresponding p-values for linear model of disease factors predicting the z-scored mean of gradient 1 at each subcortical ROI

|  | **I-Hippocampus** | | | |  |  | **C-Hippocampus** | | | |
| --- | --- | --- | --- | --- | --- | --- | --- | --- | --- | --- |
|  | **Laterality** | **MTS** | **BTCS** | **Duration** |  |  | **Laterality** | **MTS** | **BTCS** | **Duration** |
| **β** | **-0.0197** | -0.0032 | -0.0035 | -0.0003 |  | **β** | -0.0082 | 0.0004 | 0.0014 | -0.0002 |
| **p-value** | **0.004** | 0.665 | 0.631 | 0.332 |  | **p-value** | 0.151 | 0.955 | 0.828 | 0.323 |
|  |  |  |  |  |  |  |  |  |  |  |
|  | **I-Amygdala** | | | |  |  | **C-Amygdala** | | | |
|  | **Laterality** | **MTS** | **BTCS** | **Duration** |  |  | **Laterality** | **MTS** | **BTCS** | **Duration** |
| **β** | -0.0133 | 0.0027 | -0.0002 | 0.0002 |  | **β** | -0.0036 | 0.0107 | 0.002 | 0.0002 |
| **p-value** | 0.164 | 0.799 | 0.983 | 0.655 |  | **p-value** | 0.637 | 0.206 | 0.811 | 0.554 |
|  |  |  |  |  |  |  |  |  |  |  |
|  | **I-Thalamus** | | | |  |  | **C-Thalamus** | | | |
|  | **Laterality** | **MTS** | **BTCS** | **Duration** |  |  | **Laterality** | **MTS** | **BTCS** | **Duration** |
| **β** | -0.0041 | -0.0025 | -0.0122 | -0.0005 |  | **β** | 0.0041 | 0.0004 | -0.0078 | -0.0003 |
| **p-value** | 0.58 | 0.757 | 0.135 | 0.121 |  | **p-value** | 0.626 | 0.968 | 0.392 | 0.39 |
|  |  |  |  |  |  |  |  |  |  |  |
|  | **I-Caudate** | | | |  |  | **C-Caudate** | | | |
|  | **Laterality** | **MTS** | **BTCS** | **Duration** |  |  | **Laterality** | **MTS** | **BTCS** | **Duration** |
| **β** | 0.0096 | -0.0034 | **0.029** | 0.0006 |  | **β** | 0.0046 | 0.0005 | 0.0159 | 0.0008 |
| **p-value** | 0.351 | 0.765 | **0.012** | 0.13 |  | **p-value** | 0.652 | 0.962 | 0.161 | 0.071 |
|  |  |  |  |  |  |  |  |  |  |  |
|  | **I-Putamen** | | | |  |  | **C-Putamen** | | | |
|  | **Laterality** | **MTS** | **BTCS** | **Duration** |  |  | **Laterality** | **MTS** | **BTCS** | **Duration** |
| **β** | 0.0122 | -0.0003 | 0.0058 | 0.0004 |  | **β** | 0.0067 | 0.0003 | 0.0005 | 0.0002 |
| **p-value** | 0.047 | 0.96 | 0.377 | 0.119 |  | **p-value** | 0.371 | 0.972 | 0.953 | 0.567 |
|  |  |  |  |  |  |  |  |  |  |  |
|  | **I-Pallidum** | | | |  |  | **C-Pallidum** | | | |
|  | **Laterality** | **MTS** | **BTCS** | **Duration** |  |  | **Laterality** | **MTS** | **BTCS** | **Duration** |
| **β** | 0.002 | 0.0076 | -0.0083 | -0.0002 |  | **β** | -0.0063 | 0.0021 | -0.0089 | -0.0001 |
| **p-value** | 0.674 | 0.152 | 0.113 | 0.39 |  | **p-value** | 0.423 | 0.81 | 0.299 | 0.697 |

**Supplementary Table 3 –** Regression coefficients and corresponding p-values for linear models of disease factors predicting the z-scored variance of gradient 1 at each subcortical ROI

|  | **I-Hippocampus** | | | |  |  | **C-Hippocampus** | | | |
| --- | --- | --- | --- | --- | --- | --- | --- | --- | --- | --- |
|  | **Laterality** | **MTS** | **BTCS** | **Duration** |  |  | **Laterality** | **MTS** | **BTCS** | **Duration** |
| **β** | **-0.0034** | -0.0027 | -0.0005 | -4.04E-05 |  | **β** | -0.0027 | -0.0024 | 0.0004 | -7.75E-05 |
| **p-value** | **0.01** | 0.058 | 0.705 | 0.435 |  | **p-value** | 0.041 | 0.108 | 0.789 | 0.146 |
|  |  |  |  |  |  |  |  |  |  |  |
|  | **I-Amygdala** | | | |  |  | **C-Amygdala** | | | |
|  | **Laterality** | **MTS** | **BTCS** | **Duration** |  |  | **Laterality** | **MTS** | **BTCS** | **Duration** |
| **β** | -0.0033 | -0.0026 | -0.0024 | -0.0001 |  | **β** | -0.0025 | -0.0019 | -0.0005 | -8.04E-05 |
| **p-value** | 0.034 | 0.132 | 0.152 | 0.087 |  | **p-value** | 0.063 | 0.211 | 0.757 | 0.137 |
|  |  |  |  |  |  |  |  |  |  |  |
|  | **I-Thalamus** | | | |  |  | **C-Thalamus** | | | |
|  | **Laterality** | **MTS** | **BTCS** | **Duration** |  |  | **Laterality** | **MTS** | **BTCS** | **Duration** |
| **β** | -0.0032 | -0.0028 | 0.0013 | -7.32E-05 |  | **β** | -0.0024 | -0.0028 | 0.0011 | -8.64E-05 |
| **p-value** | 0.029 | 0.088 | 0.431 | 0.213 |  | **p-value** | 0.151 | 0.126 | 0.554 | 0.199 |
|  |  |  |  |  |  |  |  |  |  |  |
|  | **I-Caudate** | | | |  |  | **C-Caudate** | | | |
|  | **Laterality** | **MTS** | **BTCS** | **Duration** |  |  | **Laterality** | **MTS** | **BTCS** | **Duration** |
| **β** | -0.001 | -0.0013 | 6.35E-05 | -2.33E-05 |  | **β** | -0.0015 | -0.0014 | 0.0006 | -2.59E-05 |
| **p-value** | 0.397 | 0.333 | 0.961 | 0.627 |  | **p-value** | 0.189 | 0.255 | 0.63 | 0.571 |
|  |  |  |  |  |  |  |  |  |  |  |
|  | **I-Putamen** | | | |  |  | **C-Putamen** | | | |
|  | **Laterality** | **MTS** | **BTCS** | **Duration** |  |  | **Laterality** | **MTS** | **BTCS** | **Duration** |
| **β** | -0.002 | -0.0014 | 0.0005 | -2.81E-05 |  | **β** | -0.0018 | -0.0015 | 0.0001 | -7.69E-05 |
| **p-value** | 0.161 | 0.379 | 0.738 | 0.623 |  | **p-value** | 0.175 | 0.316 | 0.929 | 0.161 |
|  |  |  |  |  |  |  |  |  |  |  |
|  | **I-Pallidum** | | | |  |  | **C-Pallidum** | | | |
|  | **Laterality** | **MTS** | **BTCS** | **Duration** |  |  | **Laterality** | **MTS** | **BTCS** | **Duration** |
| **β** | -0.002 | -0.0019 | -0.0003 | -3.23E-05 |  | **β** | -0.0032 | -0.0031 | 0.0015 | -8.03E-05 |
| **p-value** | 0.055 | 0.093 | 0.783 | 0.428 |  | **p-value** | 0.033 | 0.061 | 0.347 | 0.184 |

**Supplementary Table 4 -** Regression coefficients and corresponding p-values for linear models of disease factors predicting the global variance of gradient 1 across all subcortical ROIs

|  | **Global Variance** | | | |
| --- | --- | --- | --- | --- |
|  | **Laterality** | **MTS** | **BTCS** | **Duration** |
| **β** | -0.8413 | -0.9904 | 0.3233 | -0.0155 |
| **p-value** | 0.086 | 0.069 | 0.542 | 0.427 |

**Supplementary Table 5 -** Bhattacharyya Distance and corresponding p-value between the 2D distribution generated by gradient 1 and gradient 2 in L-TLE and R-TLE subjects in the ipsilateral hippocampus, computed using different similarity metrics and dimensionality reduction approaches for gradient estimation. DM: Diffusion mapping. LE: Laplacian embedding. PCA: principal component analysis.

| **Method** | **Bhattacharyya Distance** | **p-value** |
| --- | --- | --- |
| **Cosine-DM** | **0.107963972** | **0.010989011** |
| **Gaussian-DM** | **0.095780277** | **0.020979021** |
| **Norm. Angle-DM** | **0.106681173** | **0.01998002** |
| **Pearson-DM** | **0.097407341** | **0.018981019** |
| **Spearman-DM** | **0.065434815** | **0.03996004** |
| Cosine-LE | 0.02770632 | 0.087912088 |
| **Gaussian-LE** | **0.034955792** | **0.042957043** |
| **Norm. Angle-LE** | **0.044388407** | **0.047952048** |
| Pearson-LE | 0.025584961 | 0.116883117 |
| Spearman-LE | 0.022037481 | 0.084915085 |
| **Cosine-PCA** | **0.1069308** | **0.022977023** |
| Gaussian-PCA | 0.059444441 | 0.06993007 |
| **Norm. Angle-PCA** | **0.111091021** | **0.016983017** |
| **Pearson-PCA** | **0.10620882** | **0.016983017** |
| Spearman-PCA | 0.031851973 | 0.228771229 |

**
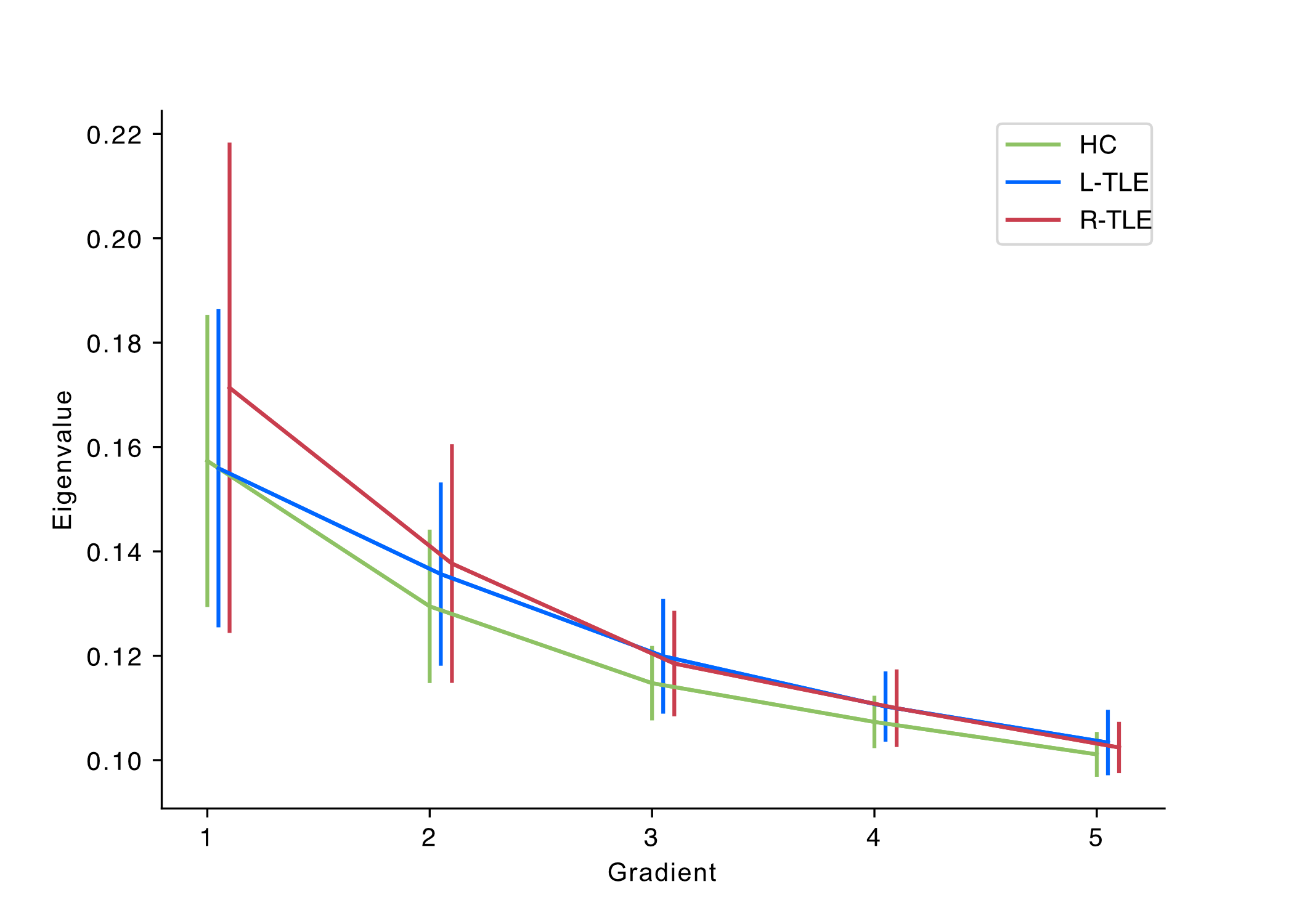
**

**Supplementary Figure 1 - Eigenvalue as a function of gradient number for healthy controls, L-TLE and R-TLE:** Mean and standard deviation of the eigenvalue corresponding to each gradient number. Differences between groups are not statistically significant for any gradient.

**
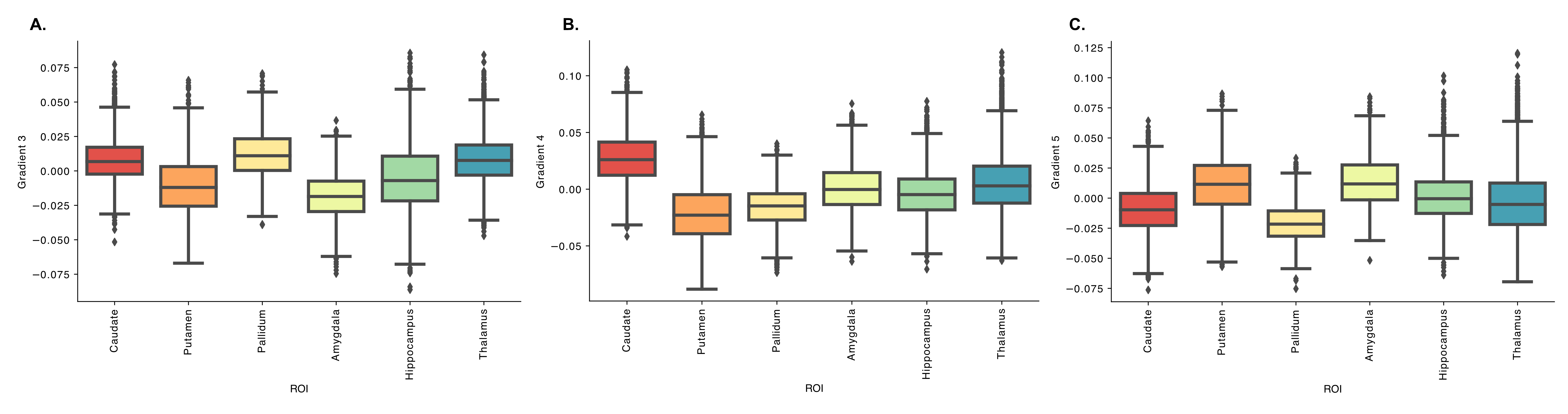
**

**Supplementary Figure 2 – Subcortical functional gradients 3, 4 and 5 across ROIs for TLE subjects:** Boxplots representing the distribution across ROIs for gradient 3 (**A.**), gradient 4 (**B.**), and gradient 5 (**C.**).


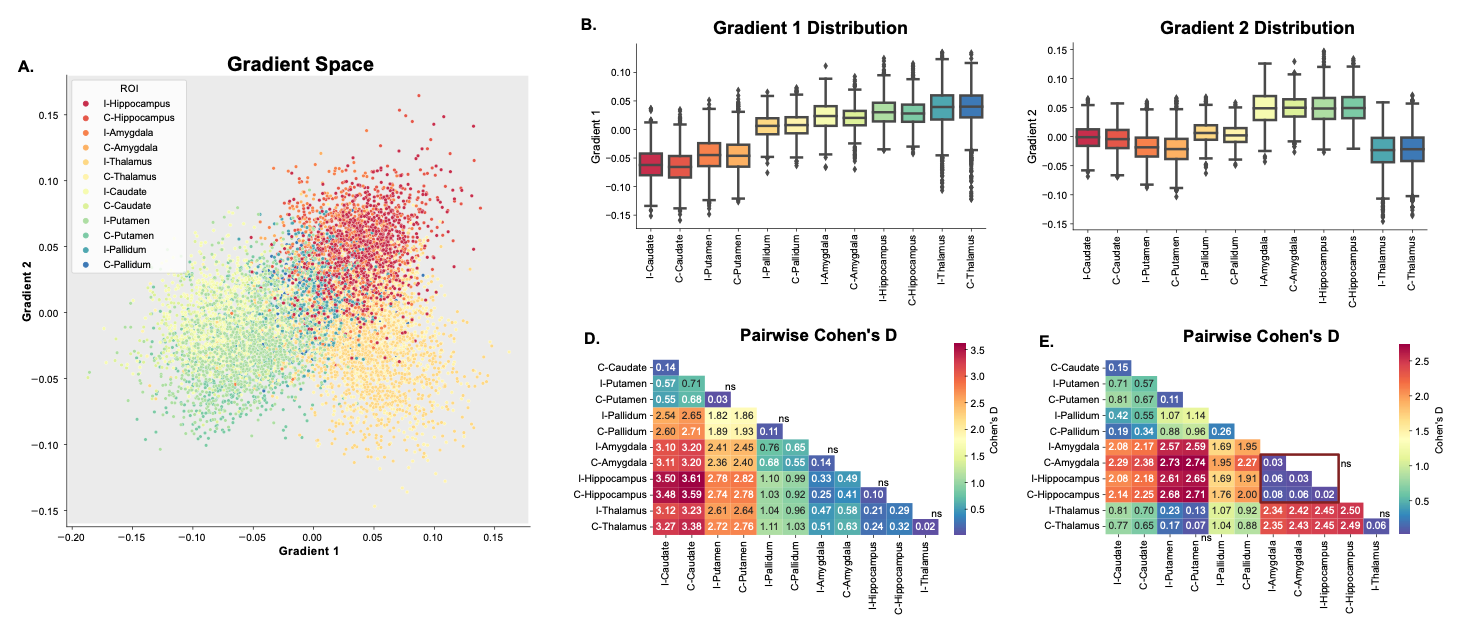


**Supplementary Figure 3 - Overview of the Subcortical Functional Gradient Across Ipsilateral and Contralateral ROIs: A.** Average gradient space generated by principal gradient 1 and 2 across all TLE subjects. Different colors represent different subcortical regions of interest (ROIs). **B.,C.** Boxplots represent the distribution across ROIs for gradient 1 (**B.**) and gradient 2 (**C.**). **D.**, **E.** Pairwise Cohen’s D values between each ROI for gradient 1 (**D.**) and gradient 2 (**E.**). Differences between ROIs were statistically significant (p_FDR_ < 0.05) unless specified otherwise (n.s.).


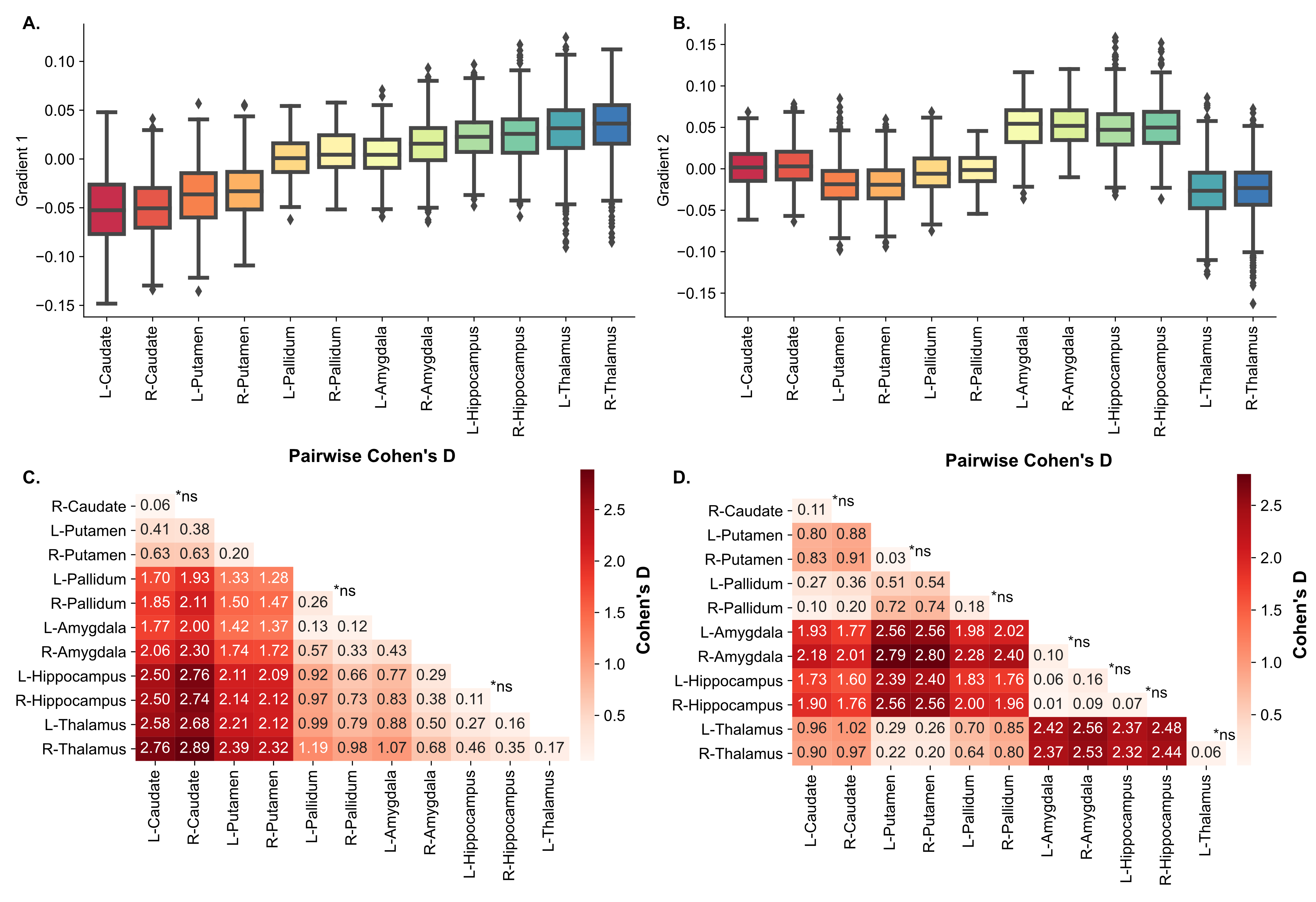


**Supplementary Figure 4 - Overview of the Subcortical Functional Gradient Across Left and Right ROIs in Control subjects: A.,B.** Boxplots represent the distribution across ROIs for gradient 1 (**A.**) and gradient 2 (**B.**). **C.**, **D.** Pairwise Cohen’s D values between each ROI for gradient 1 (**C.**) and gradient 2 (**D.**). Differences between ROIs were statistically significant (p_FDR_ < 0.05) unless specified otherwise (n.s.).

**
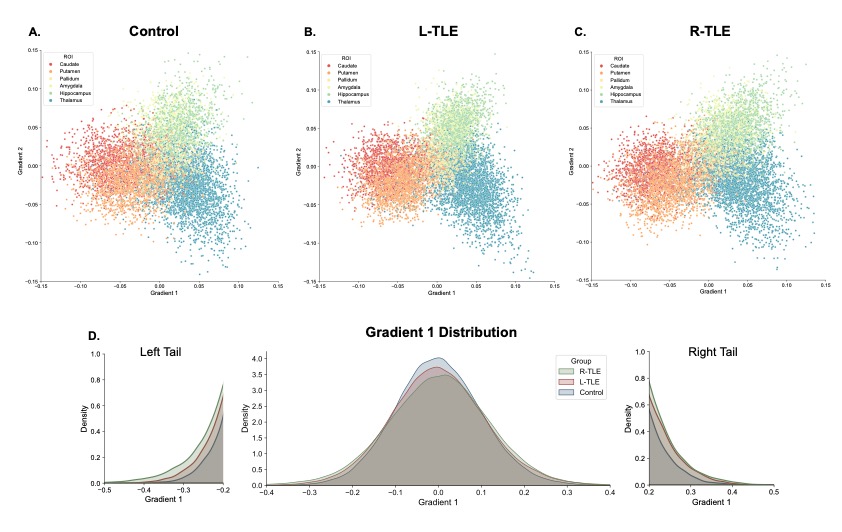
**

**Supplementary Figure 5 - Overview of the Subcortical Functional Gradient Across ROIs in Healthy Controls, L-TLE and R-TLE: A-C.** Average gradient space generated by principal gradient 1 and 2 across all **A.** control subjects, **B.** L-TLE subjects and **C.** R-TLE subjects. Ipsilateral and contralateral structures are assigned the same color in this representation. **D.** Distribution of Gradient 1 across all subjects and ROIs for each subgroup. The left and right tail show an expansion of gradient 1 for left and right TLE relative to controls.


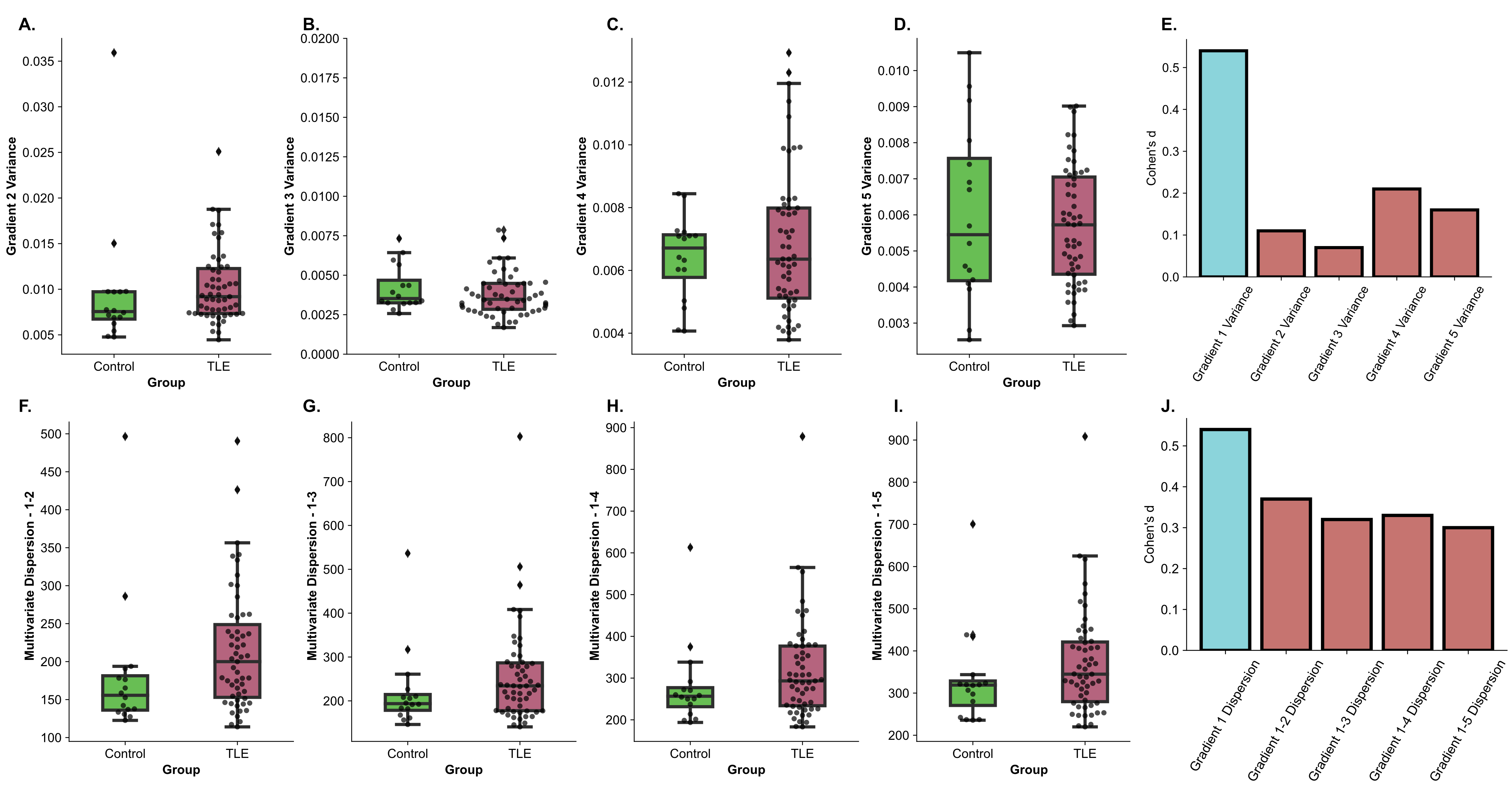


**Supplementary Figure 6 – Variance and dispersion across gradients 2-5 between TLE and controls: A.-D.** Each panel represents the variance across gradient (A.) 2, (B.) 3, (C.) 4 and (D.) 5 between control and TLE subjects. Panel **E.** shows the Cohen’s *d* between the variance of TLE and control subjects for the of each of the gradients (including gradient 1, in blue, from main text Figure 4). **F.-I.** Each panel shows the multivariate dispersion across gradients (**F.**) 1 through 2, (**G.**) 1 through 3, (**H.**) 1 through 4, and (**I.**) 1 through 5. Panel **J.** shows the Cohen’s *d* between the multivariate dispersion of TLE and control subjects, as well as the univariate dispersion/variance Cohen’s *d* (note that in the univariate case dispersion and variance are equivalent). None of the differences are statistically significant.

**
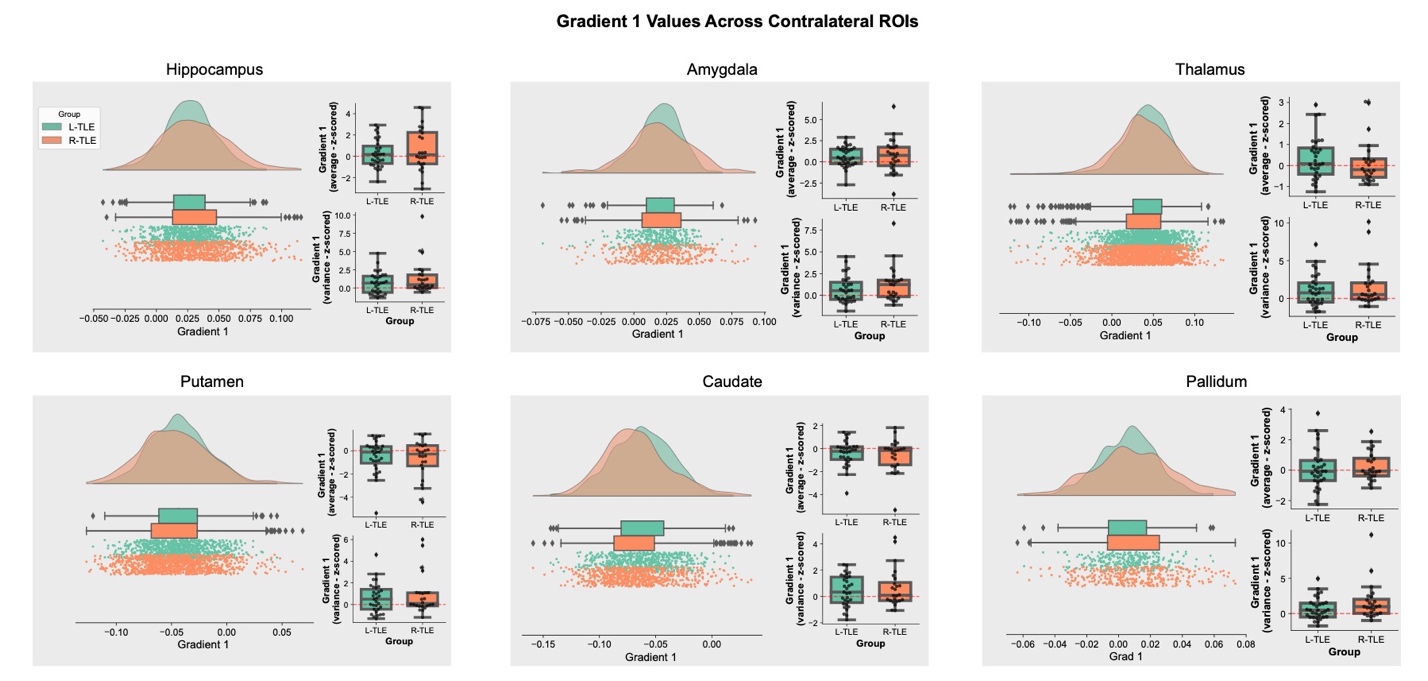
**

**Supplementary Figure 7 - Principal Gradient 1 Across Contralateral Subcortical ROIs: A-F.** Each panel represents a different subcortical ROI, and they show both, the average distribution in gradient space for gradient 1 across subjects in each group (left), and the distribution of individual gradient 1 mean and variance for subjects in each group (right). The individual subject mean and variance were z-scored relative to the distribution of gradient 1 mean and variance for controls in the same ROI, but across bilateral regions.


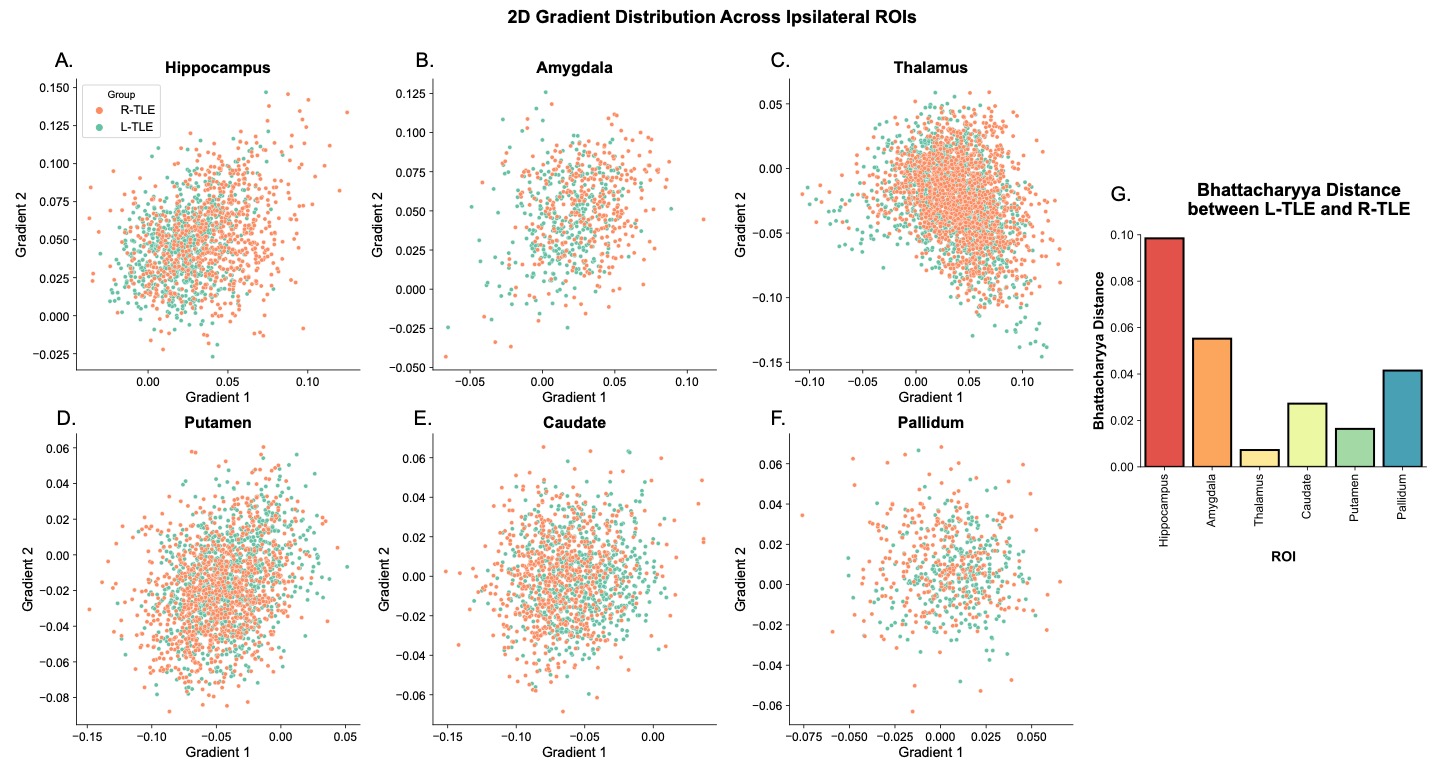


**Supplementary Figure 8** – **2-Dimensional Gradient Distribution Across Ipsilateral ROIs:** **A-F.** Group average 2-dimensional distribution generated by subcortical functional gradient 1 and 2 of R-TLE and L-TLE across ipsilateral ROIs. **G.** Bhattacharyya distance between the distribution of L-TLE and R-TLE across ROIs.

**
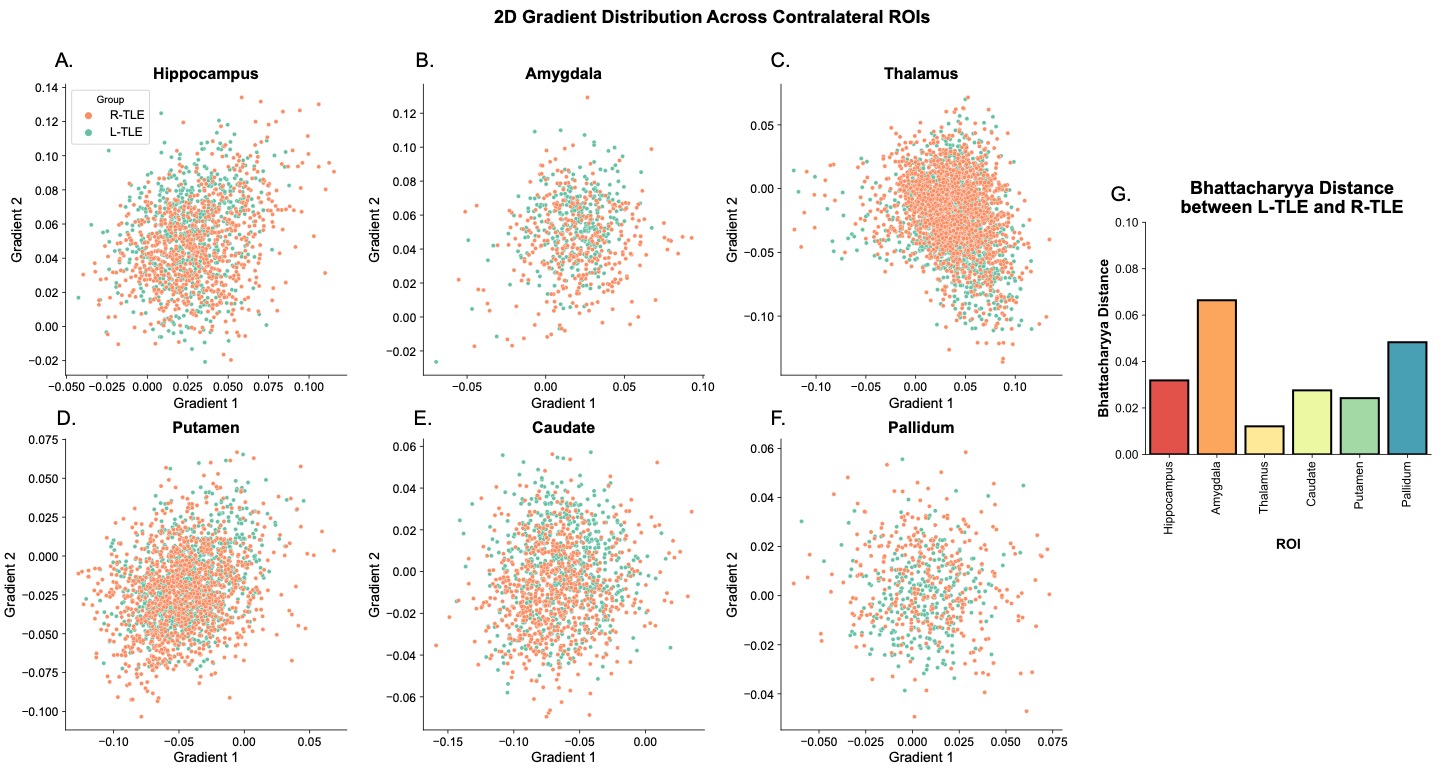
**

**Supplementary Figure 9** – **2-Dimensional Gradient Distribution Across Contralateral ROIs:** **A-F.** Group average 2-dimensional distribution generated by subcortical functional gradient 1 and 2 of R-TLE and L-TLE across contralateral ROIs. **G.** Bhattacharyya distance between the distribution of L-TLE and R-TLE across ROIs.

*Subcortical functional connectivity gradients were stable across different gradient estimation approaches*

Results for the correlation between the gradients across all ROIs computed using different methodologies are shown in Supplementary Figure 10. Across the different gradient estimation approaches, we found a large correlation across methodologies for gradient 1, with the lowest correlation between approaches that used Laplacian embedding as the dimensionality reduction technique, and approaches that did not (Supplementary Figure 10C). For gradient 2 (Supplementary Figure 10D) we found a similar pattern, with an even lower correlation between approaches that used Laplacian embedding as the dimensionality reduction technique, and approaches that did not. We also repeated the Bhattacharyya distance analysis within the ipsilateral hippocampus across all methodologies, and the findings remained statistically significant, with consistent distances, for across all approaches that used a diffusion mapping dimensionality reduction. For Laplacian embedding dimensionality reduction, cosine similarity, Pearson correlation, and Spearman correlation similarities did not produce significant findings, and had very low distances. Finally, for PCA dimensionality reduction, Gaussian kernel and Spearman similarity also had low distances that were not significant. These findings are summarized in Supplementary Table 5.


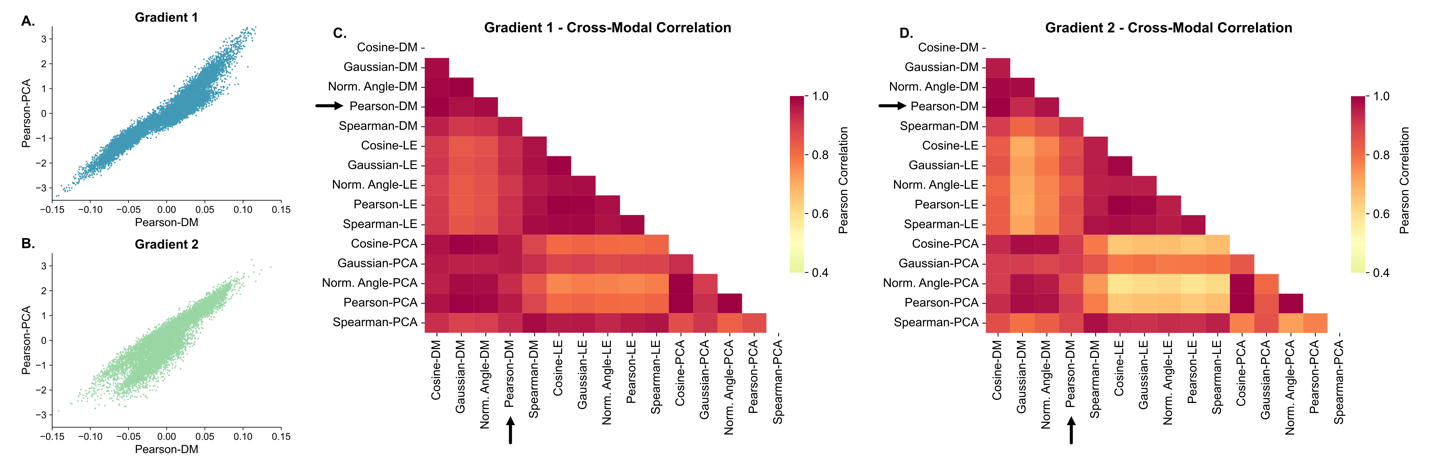


**Supplementary Figure 10 – Gradient Stability Across Estimation Methods: A.** Scatterplot between gradient 1 estimated with a Pearson correlation similarity matrix and diffusion mapping dimensionality reduction (the original approach used in this study), and gradient 1 estimated with a Pearson correlation similarity matrix and principal component analysis dimensionality (PCA) dimensionality reduction. **B.** Same as **A.** but with gradient 2. **C-D.** Absolute value of the Pearson correlation between gradient 1 (**C.**) and gradient 2 (**D.**) for different methods of estimating the similarity matrix and the subsequent dimensionality reduction. Arrows point to the rows and columns corresponding to the Pearson-DM approach used in the main findings of this study. DM – Diffusion mapping, LE – Laplacian embedding, PCA – Principal component analysis.
